# The interplay between microbiota and brain-gut axis in epilepsy treatment

**DOI:** 10.3389/fphar.2024.1276551

**Published:** 2024-01-26

**Authors:** Hanxiao Zhu, Wei Wang, Yun Li

**Affiliations:** ^1^ Department of Neurology, The First Affiliated Hospital of Dali University, Dali, China; ^2^ Clinical Medical School, Dali University, Dali, China; ^3^ Neurobiology Laboratory, China Agricultural University, Beijing, China

**Keywords:** epilepsy, brain-gut-microbiota axis, viscerosensory pathway, endocrine pathway, immune pathway

## Abstract

The brain-gut axis plays a vital role in connecting the cognitive and emotional centers of the brain with the intricate workings of the intestines. An imbalance in the microbiota-mediated brain-gut axis extends far beyond conditions like Irritable Bowel Syndrome (IBS) and obesity, playing a critical role in the development and progression of various neurological disorders, including epilepsy, depression, Alzheimer’s disease (AD), and Parkinson’s disease (PD). Epilepsy, a brain disorder characterized by unprovoked seizures, affects approximately 50 million people worldwide. Accumulating evidence suggests that rebuilding the gut microbiota through interventions such as fecal microbiota transplantation, probiotics, and ketogenic diets (KD) can benefit drug-resistant epilepsy. The disturbances in the gut microbiota could contribute to the toxic side effects of antiepileptic drugs and the development of drug resistance in epilepsy patients. These findings imply the potential impact of the gut microbiota on epilepsy and suggest that interventions targeting the microbiota, such as the KD, hold promise for managing and treating epilepsy. However, the full extent of the importance of microbiota in epilepsy treatment is not yet fully understood, and many aspects of this field remain unclear. Therefore, this article aims to provide an overview of the clinical and animal evidence supporting the regulatory role of gut microbiota in epilepsy, and of potential pathways within the brain-gut axis that may be influenced by the gut microbiota in epilepsy. Furthermore, we will discuss the recent advancements in epilepsy treatment, including the KD, fecal microbiota transplantation, and antiseizure drugs, all from the perspective of the gut microbiota.

## 1 Introduction

Epilepsy is a neurological disease characterized by recurrent, unprovoked epileptic seizures, temporary neurological dysfunction, and a host of other adverse effects (Fisher et al., 2014; Thijs et al., 2019). Seizures are transient clinical manifestations arising from synchronized, high-frequency, or abnormal excessive neuronal activity in the central nervous system (CNS). They can stem from a diverse range of etiologies associated with congenital or acquired brain malformations, structural injuries or lesions, and other brain diseases or disorders (Fisher et al., 2017a; Balestrini et al., 2021; Zhu et al., 2023). Recent study has revealed that the prevalence of active epilepsy worldwide stands at 6.38 per 1,000 individuals, whereas the lifetime prevalence is estimated at 7.60 per 1,000 individuals (Fiest et al., 2017). The annual incidence rate of epilepsy is 6.144 per 10,000 people, with an alarming long-term recurrence rate of 83.6% within a decade (Beretta et al., 2017; Fiest et al., 2017; Zhu et al., 2022). Moreover, it has been found to be linked with patient stigmatization, concurrent neurological and psychiatric disorders, as well as exorbitant medical expenses, all of which significantly impair the patient’s wellbeing and quality of life, especially in low-income nations.

The elaborate relationship between the brain and the gut is commonly referred to as the “brain-gut axis,” serving as the central nexus that connects the cognitive and emotional centers in the brain to gut function. This connection begins during development and persists throughout a person’s lifetime (Borre et al., 2014; Furness et al., 2014). Recent advancements in research have expanded our understanding of the brain-gut axis beyond its role in functional gastrointestinal disorders. Furthermore, these studies are dedicated to elucidating the impact of gut dysbiosis on the structure and function of this axis. The “brain-gut-microbiota axis” has been coined to describe this form of gut microbial involvement in brain-gut communication (Cryan and Dinan, 2012). Gut microbiota influence brain-gut interactions primarily through nerve, endocrine, immune and metabolic signaling mechanisms, spanning early life stages to neurodegenerative pathologies, and from the gut lumen to the CNS (Quigley, 2017).

Dysfunction within the brain-gut-microbiota axis may contribute to the pathogenesis and pathophysiology of gastrointestinal disorders such as IBS and obesity. Additionally, it also plays a role in the development of numerous neurological disorders, including epilepsy, depression, AD, autism spectrum disorder (ASD), Parkinson’s disease, and more (Amaral et al., 2008; Mayer, 2011; Cryan and Dinan, 2012; Park et al., 2013; Sampson et al., 2016; Vuong and Hsiao, 2017; Ding et al., 2021). In recent years, numerous studies have provided evidence supporting a close connection between the gut and epilepsy, both in clinical observations and animal experiments (Peng et al., 2018; Zhang et al., 2018; Lindefeldt et al., 2019; Gong et al., 2020; Citraro et al., 2021; Ding et al., 2021; Eor et al., 2021; Lee et al., 2021). For instance, significant differences in fecal microbial composition have been observed between individuals with epilepsy and healthy subjects, as well as before and after KD treatment in epilepsy patients and in animal models of epilepsy (Lindefeldt et al., 2019; Gong et al., 2020; Eor et al., 2021; Lee et al., 2021).

Understanding the interactions between the brain and the gut in individuals with epilepsy holds immense significance in unraveling the mechanisms of communication between these vital centers of activity in the body and the underlying pathophysiology of epilepsy. This exploration not only has the potential to identify novel therapeutic targets for epilepsy treatment but also strives to develop tailored preventive strategies for this condition. Numerous previous reviews have delved into the connection between gut microbiota and epilepsy (De Caro et al., 2019a; Dahlin and Prast-Nielsen, 2019; Fan et al., 2019; Ułamek-Kozioł et al., 2019; Holmes et al., 2020; Amlerova et al., 2021; Chatzikonstantinou et al., 2021; Ding et al., 2021; Tang et al., 2021; Arulsamy and Shaikh, 2022; Fusco et al., 2022; Iannone et al., 2022; Russo, 2022; Yue et al., 2022; Özcan et al., 2022; Wang et al., 2023), but this paper stands out as the most comprehensive analysis to date. We have curated an extensive body of clinical and animal evidence that supports the involvement of the brain-gut-microbiota axis in epilepsy and drug-resistant epilepsy. Furthermore, we meticulously outline potential pathways through which gut flora mediate epilepsy pathology. Our primary focus is on elucidating the mechanisms by which gut microbiota can be harnessed for epilepsy therapy, thereby contributing to a more profound comprehension of this critical aspect of the condition.

## 2 The close relationship between the gut microbiota and epilepsy

Several population-based studies have revealed differences in gut microbiota between individuals with epilepsy and healthy controls, albeit in relatively limited sample sizes. In a bi-directional Mendelian randomization study, researchers explored the causal relationship and identified specific gut microbe taxa associated with epilepsy, using genome-wide association study (GWAS) data of epilepsy, gut microbiota, and gut microbiota-dependent metabolites such as trimethylamine N-oxide and its predecessors. Following multiple-testing correction, the study identified a suggestive association of host-genetic-driven increase in class Melainabacteria with a lower risk of generalized epilepsy with tonic-clonic seizures, class Betaproteobacteria and order Burkholderiales with a lower risk of juvenile myoclonic epilepsy, and family Veillonellaceae with a higher risk of childhood absence epilepsy (Ouyang et al., 2022).

In order to gain a better understanding of the changes in gut microbiota in children with focal epilepsy before and after drugs treatment, Zhou et al. conducted a study that involved dividing 10 children with newly diagnosed focal epilepsy into a pre-treatment subgroup and a post-treatment subgroup (treated with oral oxcarbazepine), and included 14 healthy children of the same age as controls. The study revealed significant differences in α diversity between the pre-treatment subgroup and the control group. The relative abundance of Actinobacteria phylum, and *Escherichia*/*Shigella*, *Collinsella*, *Streptococcus*, and *Megamonas* genus was notably higher in the pre-treatment subgroup, while *Faecalibacterium* and *Anaerostipes* were enriched in the control group. After 3 months of antiseizure treatment, no significant differences were found in α diversity between the post-treatment and pre-treatment subgroups. Additionally, the differences in bacterial composition between the post-treatment children and controls were not significant. However, the relative abundance of Actinobacteria phylum notably declined after treatment compared to the pre-treatment subgroup, and the proportion of certain genera, such as *Escherichia*/*Shigella*, *Collinsella*, *Streptococcus*, and *Megamonas*, also decreased significantly (Zhou et al., 2022). Similarly, Gong S.Z. et al. also found higher levels of Actinobacteria phylum and the genus *Escherichia*/*Shigella*, *Collinsella*, *Streptococcus*, and *Megamonas* in children with focal epilepsy. After treatment with Oxcarbazepine, patients exhibited lower levels of Actinobacteria phylum and the genus *Escherichia*/*Shigella*, *Collinsella*, *Streptococcus*, and *Megamonas* (Gong, et al., 2022). These findings were consistent with the results of the study by Zhou et al. (2022).

In a separate study examining the gut microbiota composition, 30 patients with idiopathic focal epilepsy and 10 healthy subjects were analyzed (Şafak et al., 2020). Patients with epilepsy exhibited higher levels of the Proteobacteria phylum, which included genera such as *Campylobacter*, *Delftia*, *Lautropia*, *Haemophilus*, and *Neisseria*. Conversely, the healthy controls had lower abundance of this phylum. The *Clostridium* phylum, specifically *Leptotrichia* and *Fusobacterium*, were found in 10.6% of epileptic patients but were absent in the healthy controls. Moreover, the levels of the Firmicutes, Actinobacteria, and Bacteroidetes phylum were higher in the healthy control group compared to epileptic patients. At the genus level, *Blautia*, *Coprococcus*, *Faecalibacterium* and *Ruminococcus* genus of the Firmicutes phylum, *Bifidobacterium* and *Collinsellagenus* of the Actinobacteria phylum, *Bacteroides* and *Parabacteroides* genus of the Bacteroidetes phylum were significantly higher in the healthy volunteer group than in the epilepsy group (Şafak et al., 2020).

Furthermore, Huang et al. conducted a study to investigate the gut microbiota characteristics in 25 children with both cerebral palsy and epilepsy, comparing them with 21 healthy children. The results revealed that pediatric patients with cerebral palsy and epilepsy exhibited significantly higher microbial diversity and enrichment of *Bifidobacterium*, *Streptococcus*, *Akkermansia*, *Enterococcus*, *Prevotella*, *Veillonella*, *Rothia*, and *Clostridium IV* genera, while there was a noticeable reduction in *Bacteroides*, *Faecalibacterium*, *Blautia*, *Ruminococcus*, *Roseburia*, *Anaerostipes*, and *Parasutterella* genera (Huang et al., 2019). Moreover, a separate study by Liu et al. (2023) found that neonates aged 0–2 years with epilepsy showed a lower abundance of Proteobacteria phylum and a higher abundance of *Bifidobacterium* genus. These findings, in conjunction with the data presented in Tables 1, 2, suggest a potential association between epilepsy and alterations in gut microbiota. Notably, these studies have too few subjects and risk for over fitting if the data. It is crucial for future research to utilize larger and more diverse samples in order to ensure the generalizability of the findings. Additionally, researchers should employ appropriate statistical techniques to avoid overfitting and ensure the robustness of their results.

**TABLE 1:** Alterations in gut microbiota in patients with epilepsy.

Participants	Age (years)	Treatment	Alteration in gut microbiota	References
Children with refractory epilepsy	0.5–3.2	KD	Lower alpha diversity; increased levels of Bacteroidetes phylum and decreased abundance of Firmicutes phylum	Zhang et al. (2018)
Children with therapy-resistant epilepsy	2.8–15.3	KD	No changes in alpha diversity; decreased the genus Bifidobacterium (the species *Bifidobacterium longum* and *B. adolescentis*) as well as *Eubacterium rectale* and genus *Dialister*; increased abundance in *Escherichia coli*	Lindefeldt et al. (2019)
Childhood absence epilepsy	—	—	Host-genetic-driven increase in family Veillonellaceae with a higher risk of childhood absence epilepsy	Ouyang et al. (2022)
Generalized epilepsy with tonic-clonic seizures	—	—	Host-genetic-driven increase in class Melainabacteria with a lower risk of generalized epilepsy with tonic-clonic seizures	Ouyang et al. (2022)
Juvenile myoclonic epilepsy	—	—	Host-genetic-driven increase in class Betaproteobacteria and order Burkholderiales with a lower risk of juvenile myoclonic epilepsy	Ouyang et al. (2022)
Paediatric patients with cerebral palsy and epilepsy	70.43 ± 20.93 months	—	Higher microbial diversity; incaresed genera *Bifidobacterium*, *Streptococcus*, *Akkermansia*, *Enterococcus*, *Prevotella*, *Veillonella*, *Rothia*, and *Clostridium IV*; reduced genera *Bacteroides*, *Faecalibacterium*, *Blautia*, *Ruminococcus*, *Roseburia*, *Anaerostipes*, and *Parasutterella*	Huang et al. (2019)
Children with focal epilepsy	Mean age 6.35	Oral antiepileptics	Pre-reatment vs. Control: significant differences in alpha diversity; higer Actinobacteria phylum; increased genera *Escherichia/Shigella*, *Streptococcus*, *Collinsella*, and *Megamonas*; decaresed genera *Faecalibacterium* and *Anaerostipes*; Post-treatment vs. Pre-treatment: reduced Actinobacteria phylum and genera *Escherichia/Shigella*, *Streptococcus*, *Collinsella*, and *Megamonas*	Zhou et al. (2022)
Children with cerebral palsy and epilepsy	4–14	—	*Bifidobacterium*, *Bacteroidetes*, and *Prevotella* were the top three abundant genera	Huang et al. (2022)
Infants with epilepsy	0–2	—	Lower abundance of Proteobacteria phylum and higher abundance of *Bifidobacterium* genus	Liu et al. (2023)
Children with cerebral palsy and epilepsy	3–15	Fluid diet	Liquid diet vs. General diet: significant differences in Bacteroidetes and Actinobacteria phylum; increased genera *Bifidobacterium* and *Collinsella*; decreased genera *Prevotella*, *Roseburia*, *Lactobacillus*, and *Faecalibacterium*	Huang et al. (2021)
Childhood Epilepsy	2–11	—	Higer the genera *Flavobacterium*, *Holdemania*, and *Hyphomicrobium*; the genera *Megamonas* and *Coriobacterium* were observed only in the epilepsy	Türay et al. (2023)
Children with drug refractory epilepsy	26.33 ± 12.05	—	Increased richness and diversity, and Actinobacteria phylum and the genus *Enterococcus*, *Anaerostipes*, *Bifidobacterium*, *Bacteroides*, and *Blautia*	Gong et al. (2021)
Children with drug refractory epilepsy	26.33 ± 12.05	KD	Decreased the genus *Bifidobacterium*, *Akkermansia*, *Enterococcaceae* and *Actinomyces*, and increased the genus *Subdoligranulum*, *Dialister*, *Alloprevotella*	Gong et al. (2021)
An ASD patient with a history of generalized seizures	17-year-old	KD	A reduction in Firmicutes, Bacteroidetes, and Proteobacteria phylum;	Bertuccioli et al. (2022)
An ASD patient with a history of generalized seizures	17-year-old	Monosaccharides and polyols diet	Increased alpha biodiversity; Decreased Actinobacteria, Firmicutes, Lactobacilli, and Bifidobacteria, and the Firmicutes/Bacteroidetes ratio	Bertuccioli et al. (2022)
Children with epilepsy	3–16	—	Reduced trend of bacterial abundances and biodiversity; increased abundance in *Akkermansia* spp. and Proteobacteria and a decreased relative abundance in *Faecalibacterium* spp.	Ceccarani et al. (2021)
Participants with cryptogenic epilepsy	53 ± 6.72	VPA treatment	Increased the ratio of phylum Firmicutes/Bacteriodetes;	Gong et al. (2022a)
Temporal lobe epilepsy patients with anxiety disorders	31.3 ± 7.2	—	Higher abundances of Firmicutes (phylum), Proteobacteria (phylum), Lachnospirales (order), Enterobacterales (order), Lachnospiraceae (family), Enterobacteriaceae (family), Gammaproteobacteria (class), and lower abundances of Clostridia (class), *Escherichia-Shigella* (genus), and *Ruminococcus* (genus); more abundant in fungi: Saccharomycetales fam. incertae sedis (family), Saccharomycetales (order), Saccharomycetes (class), and Ascomycota (phylum)	Wei et al. (2023)
Patients with drug-resistant epilepsy	16–50	Ciprofloxacin	Increased Bacteroidetes/Firmicutes ratio	Cheraghmakani et al. (2021)
Children with cerebral palsy and epilepsy	8.8 (4.5–16.9)	—	Decreased abundances of *Bacteroides fragilis* and *Dialister invisus*; increased abundances of *Phascolarctobacterium faecium* and *Eubacterium limosum*	Peng et al. (2023)
Children with cerebral palsy and drug-resistant epilepsy	9.7 (4.5–15.4)	—	Higher abundances of *Veillonella parvula*	Peng et al. (2023)
Patients with epilepsy	31.6 ± 12.2	—	Increased the genus *Fusobacterium*, *Megasphaera*, *Alloprevotella*, and *Sutterella*	Dong et al. (2022)
Children with focal epilepsy	6 (5, 9)	—	Higher Actinobacteria phylum and the genus *Escherichia/Shigella*, *Collinsella*, *Streptococcus*, and *Megamonas*	Gong et al. (2022)
Children with focal epilepsy	6 (5, 9)	Oxcarbazepine	Lower Actinobacteria phylum Actinobacteria phylum and the genus *Escherichia/Shigella*, *Collinsella*, *Streptococcus*, and *Megamonas*	Gong et al. (2022)
Drug-resistant epilepsy patients	27.70 ± 13.32	—	Lower microbial diversity; higher the Phylum Firmicutes and the class Bacilli	Dai et al. (2022)
Rasmussen encephalitis with focal intractable seizures	18	Probiotic kefir supplementation	Increased *Lactobacillus* and *Bifidobacterium* species	Lemos et al. (2022)
Children with Intractable Epilepsy	1.16–6.92	—	Lower microbiota richness; higher abundance of Actinobacteria and lower abundance of Bacteroidetes	Lee et al. (2020)
Pediatric patients with refractory epilepsy	1.95 ± 3.10	—	Increased abundance of the Firmicutes and Proteobacteria phylum; decreased abundance of Bacteroidetes and Actinobacteria; elevated abundance of *Cronobacter*, and decreased the relative abundance of beneficial genera such as *Bacteroides*, *Prevotella*, and *Bifidobacterium*	Xie et al. (2017)
Pediatric patients with refractory epilepsy	1.95 ± 3.10	KD	Increased abundance of the *Bacteroides* and *Prevotella*; decreased abundance of the *Cronobacter*, *Erysipelatoclostridium*, *Streptococcus*, *Alistipes*, *Ruminiclostridium*, *Barnesiella* and *Enterococcus*	Xie et al. (2017)
Patients with idiopathic focal epilepsy	41.3 ± 12.2	—	Higher levels of the Proteobacteria phylum, which included genera such as *Campylobacter*, *Delftia*, *Lautropia*, *Haemophilus*, and *Neisseria*	Şafak et al. (2020)
Patients with drug- responsive epilepsy	44 ± 17.2	—	Increased relative abundance of *Bacteroides finegoldii* and *Ruminococcus_g2*	Lee et al. (2021)
Patients with drug-resistant epilepsy	41 ± 13.6	—	Increased relative abundance of *Negativicutes*	Lee et al. (2021)
Patients with drug-resistant epilepsy	28.4 ± 12.4	—	Greater microbial community richness and evenness; higher abundance of the Firmicutes phylum and relatively lower abundance of the Bacteroidetes phylum; elevated abundance of numerous rare bacteria, including *Clostridium XVIII*, *Fusobacterium*, *Methanobrevibacter*, *Atopobium*, *Holdemania*, *Delftia*, *Coprobacillus*, *Dorea*, *Saccharibacteria*, *Paraprevotella*, *Gemmiger*, *Akkermansia*, *Ruminococcus*, *Neisseria*, *Coprococcus*, *Phascolarctobacterium*, and *Roseburia*	Peng et al. (2018)
Patients diagnosed with epilepsy	26.33 ± 12.05	—	Increased levels of Actinobacteria and Verrucomicrobia phylum and decreased levels of Proteobacteria phylum; increased *Prevotella_9*, *Blautia*, *Bifidobacterium*, and others genus	Gong et al. (2020)
Patients with drug-resistant epilepsy	26.33 ± 12.05	—	Increased Actinobacteria, Verrucomicrobia, and Nitrospirae phylum and several genera including *Blautia*, *Subdoligranulum*, *Bifidobacterium*, *Dialister*, and *Anaerostipes*	Gong et al. (2020)

**TABLE 2:** Alterations in gut microbiota in animal models of epilepsy.

Animals	Age (years)	Treatment	Alteration in gut microbiota	References
Dogs with idiopathic epilepsy	1–11	—	No changes in α-diversity and *Lactobacillus* genus	Muñana et al. (2020)
Epileptic dogs	2–6	—	Reduced abundance of *Pseudomonadales*, *Pseudomonadaceae*, *Pseudomonas*, *Pseudomona_graminis*, *Peptococcaceae*, *Ruminococcaceae*, *Anaerotruncus* as well as *Prevotellaceae*	García-Belenguer et al. (2021)
Epileptic dogs	5.6 ± 1.9	—	Higher abundance of *Lactobacillus*	García-Belenguer et al. (2023)
Epileptic dogs	5.6 ± 1.9	Ketogenic medium chain triglycerides (MCT)- enriched diet	Higher abundance of *Negativicutes* and *Selenomonadales*	García-Belenguer et al. (2023)
Dogs with drug-refractory epilepsy	6.8 ± 1.8	MCT diet	Increased the abundance of Firmicutes; decreased the abundance of Bacteroidetes and Fusobacteria	García-Belenguer et al. (2023)
The PTZ-induced kindled rats	90 days	Prednisolone administration	Increased abundance of Verrucomicrobia, Actinobacteria, and *Saccharibacteria*	de Lima et al. (2022)
Chronic PTZ-kindled model rats	7–8 weeks	Anticonvulsant chemical Q808	Increased abundance of *Lactobacillus*, *Roseburia*, *Alloprevptella*, *Prevotellaceae_NK3B31_group*, *Prevotellaceae_UCG-001*, and *Prevotella_9*	Li et al. (2022)
Lithium chloride-pilocarpine-induced epilepsy rats	—	—	Reduced the genus *Helicobacter*, *Prevotellaceae_UCG-001*, and Ruminococcaceae*_UCG-005*	Gong et al. (2022b)
Rats with Lithium-Pilocarpine-Induced Temporal Lobe Epilepsy	60-day-old	—	Lower species richness; increased phylum Desulfobacterota and decreased Patescibacteria	Oliveira et al. (2022)
Neonatal rat model of infantile spasms syndrome (IS)	Four days after birth	Switching KD to a normal diet	lower abundance of the genus *Streptococcus*, *Staphylococcus* and higher *Rothia*; reduced *Streptococcus thermophilus* and *Streptococcus azizii* species; increased *Enterococcus faecium* species	Shearer et al. (2023)
Rat model of symptomatic IS	Four days after birth	KD and antibiotic administration	Increased abundance of *Streptococcus thermophilus* and *Lactococcus lactis*	Mu et al. (2022a)
WAG/Rij rats (a genetic animal model of absence epilepsy)	1, 4, and 8 months of age	—	lower Bacteroidetes/Firmicutes ratio	Citraro et al. (2021)
WAG/Rij rats (a genetic animal model of absence epilepsy)	6 months of age	FMT from Wistar non-epileptic donors	Increased *Coprococcus* (*C eutactus*) and *Phascolarctobacterium* (*P succinatutens*) genera, and decreased *Flexispira* genera	Citraro et al. (2021)
Wistar rats	6 months of age	FMT from WAG/Rij rats	Decreased the relative abundance of *C eutactus* and an increase of *Bacteroides* (*B dorei* and *B eggerthii*), *Parabacteroides* (*P distasonis*), and *Akkermansia* (*A muciniphila*)	Citraro et al. (2021)
Mouse model of refractory epilepsy	3–4 week old	KD	Decreased alpha-diversity; Increases *A. muciniphila* and *Parabacteroides*	Olson et al. (2018)
The *Kcna1* ^*−/−*^ mouse model for generalized tonic-clonic seizures	3–4 week old	KD	Increases *A. muciniphila* and *Parabacteroides*	Olson et al. (2018)
The DBA/1 mouse model of sudden unexpected death in epilepsy	—	High-tryptophan diet	Increased richness and diversity and Proteobacteria and Actinobacteria phylum	Yue et al. (2021)
Kainic acid (KA)-induced mouse model	—	Lipopolysaccharide (LPS) injections for 4 consecutive days	Increased the abundance of *Ruminococcus*	Ding et al. (2022)
Dravet mice	14–26 days after birth	—	Reduced species richness; increased ratio of Firmicutes and Bacteroidetes phylum;	Miljanovic and Potschka (2021)
Dravet mice	P14–26 days after birth	KD	Increased the abundance of Firmicutes and decreased the abundance of Bacteroidetes phylum; elevated in *Clostridium*, Oscillospira, Acetatifactor, and Enterohabdus abundance	Miljanovic and Potschka (2021)
Mouse model of trimethyltin chloride-induced epilepsy-like seizure	—	Oral gavage of chlorogenic acid	Increased abundance of *Lactobacillus* genus	Xi et al. (2022)

However, a significant portion of current research aimed at understanding the role of gut flora in epilepsy has primarily focused on patients with drug-resistant epilepsy, which refers to cases where seizures cannot be effectively controlled with antiseizure drugs. Drug-resistant epilepsy, where approximately 30%–40% of individuals with epilepsy are resistant to multiple anti-seizure drugs, presents a significant challenge in treatment (Tang et al., 2017). To investigate potential factors contributing to drug resistance, a study analyzed the gut microbiota of 42 patients with drug-resistant epilepsy, 49 with drug-sensitive epilepsy, and 65 healthy controls. The results revealed that patients with drug-resistant epilepsy showcased an altered gut microbiome composition, characterized by a pronounced increase in the abundance of various rare bacteria. In contrast, the gut microbiota of patients with drug-sensitive epilepsy closely resembled that of healthy controls (Peng et al., 2018). At a higher taxonomic level, patients with drug-resistant epilepsy exhibited greater microbial community richness and evenness. Specifically, they displayed relatively higher abundance of the Firmicutes phylum and relatively lower abundance of the Bacteroidetes phylum. Additionally, several rare phylum demonstrated an increasing trend in patients with drug-resistant epilepsy. For instance, the phylum Verrucomicrobia was also more abundant in drug-resistant epilepsy patients compared to both drug-sensitive epilepsy patients and healthy controls. Moreover, at the genus level, patients with drug-sensitive epilepsy showed significantly higher abundance of Bacteroidetes and two genera, *Bacteroides* and *Barnesiella*. In contrast, the microbial composition of drug-resistant epilepsy patients was characterized by an elevated abundance of numerous rare bacteria, including *Clostridium XVIII*, *Fusobacterium*, *Methanobrevibacter*, *Atopobium*, *Holdemania*, *Delftia*, *Coprobacillus*, *Dorea*, *Saccharibacteria*, *Paraprevotella*, *Gemmiger*, *Akkermansia*, *Ruminococcus*, *Neisseria*, *Coprococcus*, *Phascolarctobacterium*, and *Roseburia* (Peng et al., 2018). Gong et al. (2020) examined the fecal microbiota of 55 patients diagnosed with epilepsy and 46 healthy individuals to unravel the intricate interplay between gut microbiome and epilepsy. Differences were observed in the structure and composition of the fecal microbiota among patients with various clinical prognoses, as well as between patients and healthy controls. For instance, Verrucomicrobia, and Nitrospirae phylum and several genera including *Blautia*, *Subdoligranulum*, *Bifidobacterium*, *Dialister*, and *Anaerostipes* were enriched in patients with drug-resistant epilepsy. The alterations in the microbiome observed in patients with epilepsy included increased levels of Actinobacteria and Verrucomicrobia phylum, while the levels of Proteobacteria phylum were decreased. In addition, *Prevotella_9*, *Blautia*, *Bifidobacterium*, and other genera were found to be increased in epilepsy patients. Notably, the models constructed based on the gut microbiome were capable of distinguishing between drug-resistant and drug-sensitive epilepsy (Gong et al., 2020). Interestingly, an exploratory study found that patients with drug-resistant epilepsy and drug-sensitive epilepsy did not show significant differences in alpha and beta diversity analyses, but the composition of the gut microbiota did. In particular, the drug-sensitive group showed an increased relative abundance of *Bacteroides finegoldii* and *Ruminococcus_g2*, whereas the drug-resistant group showed an increased relative abundance of *Negativicutes*, a phylum belonging to the Firmicutes (Lee et al., 2021).

A comprehensive analysis of the fecal microbiota in pediatric patients with refractory epilepsy has unveiled significant differences in the diversity of their intestinal microbiota compared to age-matched healthy infants (Xie et al., 2017). For example, infants with refractory epilepsy exhibited a notable increase in the abundance of the Firmicutes and Proteobacteria phylum, while experiencing a decrease in the presence of Bacteroidetes and Actinobacteria in their fecal microbiota. Delving deeper into the microbial composition at the genus level, infants with epilepsy displayed a significant rise in the abundance of *Cronobacter*, a potential pathogenic bacterium, while concurrently experiencing a decrease in the relative abundance of beneficial genera such as *Bacteroides*, *Prevotella*, and *Bifidobacterium* (Xie et al., 2017). In addition, Lindefeldt et al. (2019) investigated the fecal microbiota of 12 children with therapy-resistant epilepsy and unraveled compelling alterations in the relative abundance of key phylum within the intestinal microbiota. The patients with epilepsy exhibited a significant decrease in the presence of the Bacteroidetes and Proteobacteria phylum. Inversely, there was a substantial increase in the relative abundance of the Actinobacteria and Firmicutes phylum. Consistently, Lee et al. conducted a study focusing on the gut microbiome of 8 children with intractable epilepsy and 32 age-matched healthy participants. The results also revealed lower microbiota richness in the epilepsy children compared to the healthy controls. The epilepsy group exhibited higher abundance of Actinobacteria and lower abundance of Bacteroidetes compared to the healthy controls. What is more, *Enterococcus faecium*, *Bifidobacterium longum*, and *Eggerthella lenta* emerged as strong potential biomarkers in the refractory epilepsy patients (Lee et al., 2020).

In addition to clinical studies, evidence from animal models of epilepsy supports the relationship between gut microbiota and the pathophysiology of epilepsy. Sprague-Dawley (SD) rats exposed to chronic restraint stress exhibited heightened vulnerability to seizures, as even a minimal amount of basolateral amygdala stimulation was sufficient to trigger complete and persistent seizures. Furthermore, when fecal contents from stressed donors were transplanted into the gastrointestinal tract of specific pathogen-free (SPF) rats, the recipients displayed an amplified susceptibility to seizure-inducing stimuli, along with prolonged seizure duration. SPF rats receiving fecal transplants from sham-stressed subjects exhibited increased seizure thresholds and shortened seizure duration. On the contrary, the implantation of intestinal microbiota from non-stressed rats into stressed animals demonstrated a noteworthy reduction in the duration of epileptic seizures within the stressed group of rats (Medel-Matus et al., 2018). The induction of intestinal inflammation through the intracolonic administration of 2,4,6-trinitrobenzene sulfonic acid (TNBS) in adult male rats resulted in an augmented susceptibility to pentylenetetrazole (PTZ) seizures, which exhibited a strong correlation with the severity and progression of intestinal inflammation (Riazi et al., 2008). Central antagonism of TNF alpha using a monoclonal antibody successfully prevented the increase in seizure susceptibility. Analysis of the gut microbiota between WAG/Rij rats (a recognized genetic model of absence epilepsy) and non-epileptic Wistar rats revealed notable differences in beta diversity and specific phylotypes across all age groups, as well as significant variations in the Bacteroidetes/Firmicutes ratio (Citraro et al., 2021). Importantly, fecal microbiota transplantation (FMT) from both Wistar and ethosuximide-treated WAG/Rij donors to WAG/Rij rats resulted in a significant reduction in the frequency and duration of seizures. Further, histological findings further highlighted that WAG/Rij rats displayed intestinal villi disruption and inflammatory infiltrates as early as 1 month of age, prior to the onset of seizures. FMT partially restored intestinal morphology while also significantly modifying the gut microbiota, leading to a concurrent reduction in absence seizures (Citraro et al., 2021).

Similarly, the dextran sulfate sodium (DSS)-induced intestinal inflammation in mice led to an increased susceptibility to PTZ-induced seizures (De Caro et al., 2019b). Reducing intestinal inflammation through the administration of alpha-lactoalbumin (ALAC) and sodium butyrate (NaB) exhibited significant anti-seizure effects in mice, but failed to demonstrate efficacy as anti-seizure agents in control mice without colitis at the same doses. Notably, in DSS-treated mice, the anti-seizure efficacy of valproic acid (VPA) was compromised compared to its ability to prevent seizures in non-DSS-treated mice (De Caro et al., 2019b). Then again, mice receiving microbiota from epileptic animals exhibited a higher propensity to develop status epilepticus compared to recipients of “healthy” microbiota, particularly after receiving subclinical doses of pilocarpine. This indicates an increased susceptibility to seizures influenced by the microbiota (Mengoni et al., 2021).

In brief, a wealth of clinical and animal experimental evidence (along with Tables 1, 2) supports the hypothesis of a close relationship between gut microbiota and epilepsy.

## 3 The role of gut microbiota-mediated visceral sensory pathways in epilepsy

The Enteric nervous system (ENS) is a peripheral nervous system substructure that directly controls the gastrointestinal tract and plays a vital role in the reflex physiological control of the body (Costa et al., 2000). Comprising over 100 million interconnected neurons, the ENS is a highly complex microcircuit designed to function autonomously, independent of the CNS and spinal cord (Gershon, 2010).

While the ENS is capable of autonomous behavior, it maintains bidirectional communication with the CNS. This communication is usually through the sympathetic nervous system via afferent sensory pathways in the vagus nerve and efferent motor pathways in the prevertebral ganglion, respectively (Khlevner et al., 2018). The ENS innervates visceral smooth muscle and other organs involved in gastrointestinal secretory, sensory, motor, endocrine, and immune functions. It transmits sensory signals through the spinal cord and vagus nerve to the brainstem and sensorimotor brain circuits, with modulation from affective and cognitive networks (bottom-up pathways) (Craig, 2003). Additionally, the autonomic nervous system’s sympathetic and parasympathetic efferent branches directly connect emotional arousal and autonomic brain circuits to the ENS (top-down pathway) (Mayer, 2011; Van Oudenhove et al., 2016). The brain and gastrointestinal tract have a complex bidirectional interaction to regulate normal digestive processes and integrate them with the organism’s emotional and physiological states (Craig, 2002).

**FIGURE 1:**
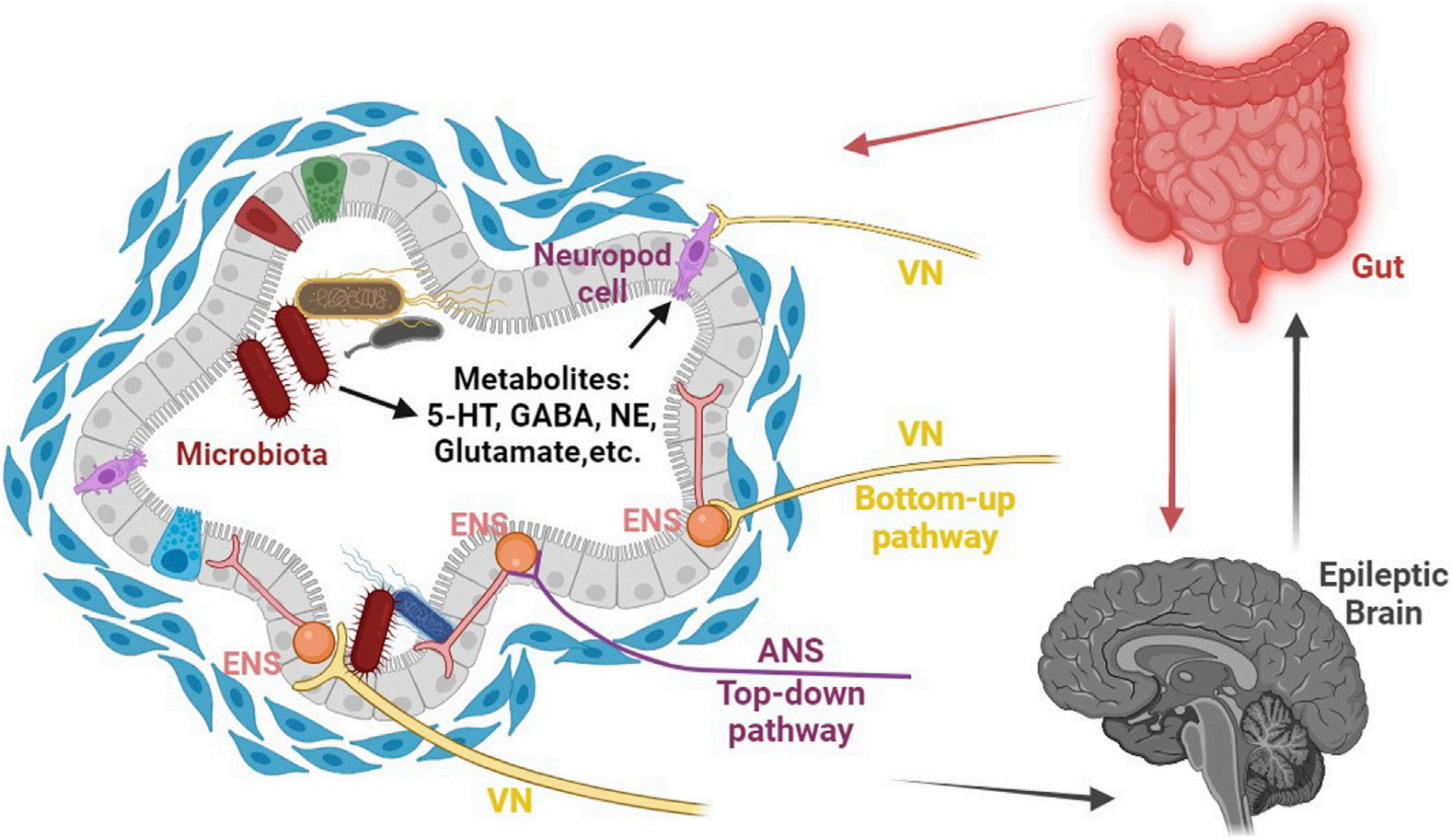
Schematic representation of the role of gut microbiota-mediated visceral sensory pathways in epilepsy. The gut microbiota possesses the remarkable capacity to directly affect the CNS through the vagus nerve, as well as indirectly by influencing the enteric nervous system (ENS) and generating essential metabolites (such as 5-HT, NE, glutamate, GABA, and more) that can traverse the blood-brain barrier, ultimately regulating epilepsy susceptibility by modulating the excitability of the CNS. Abbreviations: CNS, central nervous system; ENS, enteric nervous system; ANS, autonomic nervous system; VN, vagus nerve; NE, norepinephrine.

Interestingly, emerging evidence suggests that the gut microbiota plays a significant role in regulating the brain-gut axis. The microbiota has the ability to influence the CNS directly through the vagus nerve or indirectly by modulating the ENS (Bercik et al., 2011) (see Figure 1). On the side, an essential player in the communication between the brain and gut is 5-hydroxytryptamine (5-HT). It is present in both the ENS and CNS, acting as a crucial mediator in brain-gut communication. As a hormone throughout the loop, further highlighting its importance in this intricate system (Wikoff et al., 2009).

Remarkably, vagus nerve stimulation (VNS) has emerged as a widely utilized therapy for epilepsy since its initial discovery in 1988 (Attenello et al., 2015). The electrical stimulation of vagal afferent fibers has been found to have an effect on the brain’s concentrations of various neurotransmitters, such as 5-hydroxytryptamine, gamma-aminobutyric acid (GABA), and glutamate. This alteration in neurotransmitter levels may provide an explanation for the effectiveness of vagus nerve stimulation in treating epilepsy (Ressler and Mayberg, 2007). Besides that, recent studies have shed light on the connection between gut stimulation and modulation of brain activity through the autonomic nervous system. In mice administered live *Campylobacter* jejuni, it was observed that the c-fos gene expression is upregulated in the vagus sensory ganglia and the nucleus of the solitary tract (nTS), which serves as the primary sensory relay nucleus for the vagus. This finding suggests that gut stimulation may have the ability to influence brain activity through the autonomic nervous system (Goehler et al., 2005). Specifically, the enteroendocrine cells (EECs) can detect signals released by gut microbiota through various receptors, and interact with neurotransmitters like GABA, glutamate, 5-hydroxytryptophan, and norepinephrine, which are released by gut microbiota. The signals from the EECs are then transmitted through vagal synapses to neurons, ultimately modulating the excitability of the CNS (Wang and Powley, 2007; Kaelberer et al., 2018; Gribble and Reimann, 2019; Kuwahara et al., 2020; Yu et al., 2020) (see Figure 1). For instance, Kaelberer et al. (2018) discovered the presence of intestinal endocrine cells, known as neuropod cells, that can synapse with vagal neurons and utilize glutamate as a neurotransmitter to rapidly transmit intestinal luminal signals on a millisecond scale. These neuroepithelial circuits establish a direct connection between the gut lumen and the brainstem, creating a physical conduit for the brain to perceive gut stimuli with remarkable temporal precision and topographical resolution.

## 4 The role of gut microbiota-mediated endocrine pathways in epilepsy

### 4.1 Endocrine pathways in brain-gut signaling

The intricate mechanisms through which the brain and gut interact involve the release of endocrine mediators primarily derived from the hypothalamic-pituitary-adrenal (HPA) axis (Mayer et al., 2015). Part of the limbic system, the HPA axis is typically activated by stressors or systemic pro-inflammatory cytokines, and it primarily regulates emotional responses. Stress, in particular, induces emotional responses and stimulates hypothalamic activity. This stimulation triggers the secretion of corticotropin-releasing factor (CRF) (Stengel and Tache, 2010), which, in turn, stimulates the release of adrenocorticotropic hormone (ACTH) from the pituitary gland. ACTH then acts on the adrenal glands to promote the secretion of cortisol. Elevated levels of cortisol, a crucial stress hormone, interact with the gut to modulate various aspects of gastrointestinal function, visceral sensation, autonomic activity, and behavior (Ulrich-Lai and Herman, 2009).

At the intestinal level, these effector molecules exert effects on motility and secretion, intestinal permeability, local and systemic immune function and inflammation, as well as microbiota composition and function. For instance, increased intestinal permeability can disrupt the intestinal microbiota, resulting in elevated levels of pro-inflammatory cytokines (such as tumor necrosis factor α29) and the release of serotonin (5-HT) from enteroendocrine cells (Liebregts et al., 2007; Collins et al., 2012). These processes can enhance the sensitivity of visceral afferent nerves, amplifying stress-induced changes in motility, secretion, and permeability, which further heightens afferent signaling from the ENS (Keightley et al., 2015).

Both animal and human studies indicate that chronic or prolonged stress may lead to a sustained increase in the responsiveness of the brain’s central stress loop, ultimately resulting in functional and affective disturbances. Moreover, chronic or prolonged stress has been associated with the onset and exacerbation of symptoms in IBS (Whitehead et al., 1992). Patients with IBS often exhibit stress-induced alterations in gastrointestinal motility, gut sensation, autonomic regulation, and the response of the HPA axis (Whitehead et al., 2002). Over time, disruptions in the HPA axis can result in dysregulation of stress responses, pain modulation, and various other brain circuits, creating a maladaptive and mutually reinforcing cycle of brain-gut interactions.

Additionally, a diverse array of peptide hormones secreted by intestinal endocrine cells play a key role in regulating brain-gut signaling (Steinert et al., 2017). These intestinal peptides not only regulate gastrointestinal motility and sensitivity but also homeostatic brain circuits in the brainstem and hypothalamus through vagal afferent-terminal receptors. By doing so, they regulate food intake and energy homeostasis based on the body’s energy resources and nutritional needs. And then this regulatory neural network is involved in the motivational, emotional, and learning aspects of food reward (Alonso-Alonso et al., 2015). The combination of sensory signals from the gut, along with oral (taste) and extra-sensory inputs such as vision and odor, ultimately determines the rewarding value of food, thereby influencing appetite and feeding behavior (Weltens et al., 2014). Under normal circumstances, these gut peptides maintain a balance with the homeostatic network established by brain regions. However, when dysfunction occurs at any level of the brain-gut axis, this balance can be disrupted, leading to abnormal processing of signals, both physiological and noxious. Prolonged enhancement of afferent visceral signals may even impact mood, fear, anxiety, and other emotional processes (Berthoud and Morrison, 2008; Mayer et al., 2015).

### 4.2 Endocrine signaling between the gut microbiota and the brain plays a vital role in epilepsy

**FIGURE 2:**
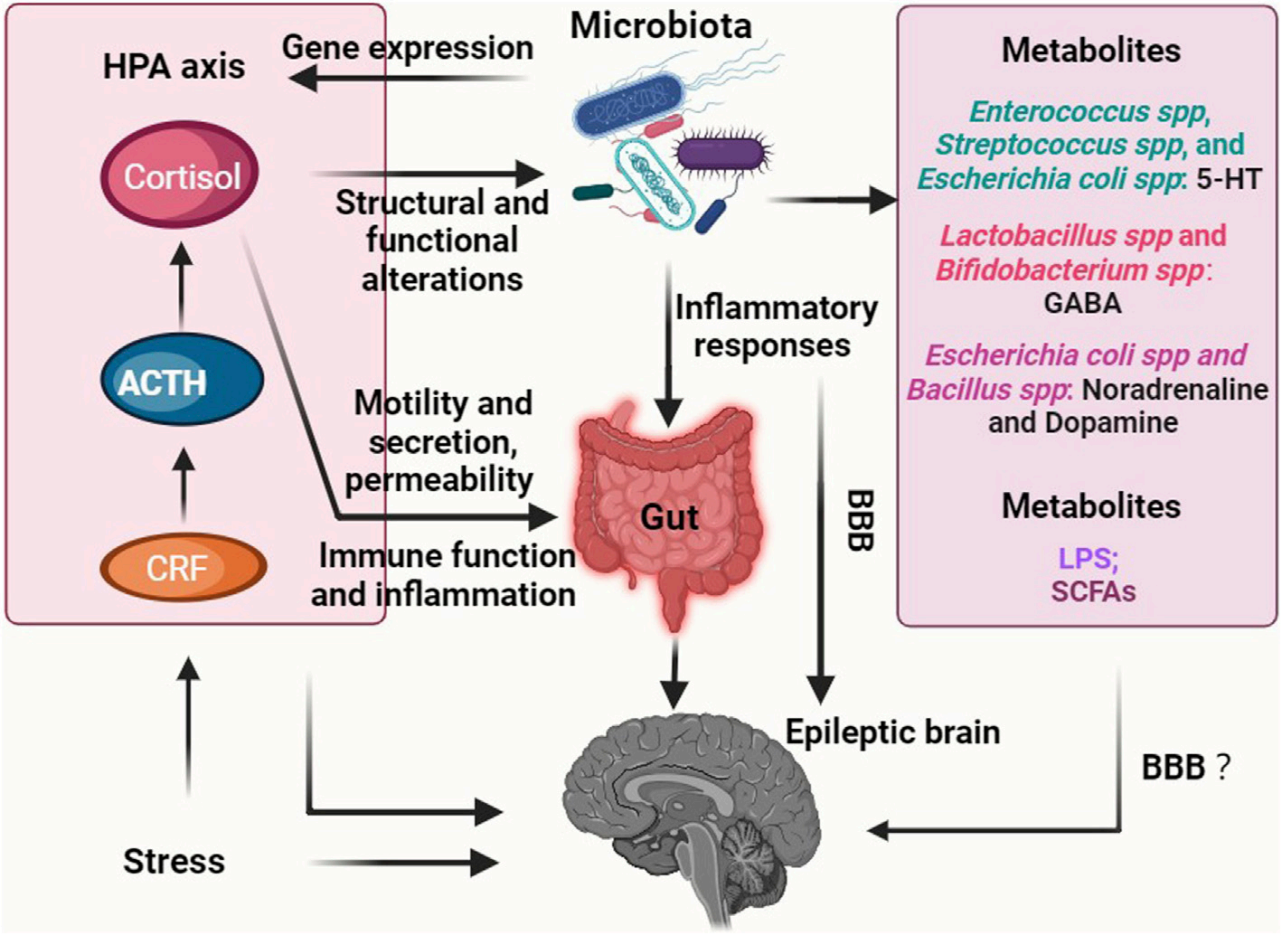
Overview of role of gut microbiota-mediated endocrine pathways in epilepsy. Chronic stress has emerged as a potential catalyst for disrupted gut microbiota, which, in turn, can upregulate the production of metabolites (such as LPS) that promote epilepsy. Additionally, these imbalances in the gut microbiota can trigger the secretion of inflammatory factors, further exacerbating the condition. Consequently, an abnormal GABA/glutamate ratio ensues, ultimately leading to the onset of epilepsy. Conversely, a healthy gut microbiota has the ability to generate beneficial metabolites like SCFAs and 5-HT, which play a crucial role in inhibiting the occurrence of epilepsy. Furthermore, the interaction between the microbiota-gut-brain axis and epilepsy involves the HPA axis, thereby highlighting its significance in this intricate relationship. Notably, the question of whether metabolites, including neurotransmitters, produced through the metabolism or regulation by the gut microbiota can permeate the BBB, and whether their effects on epilepsy are direct, indirect, or both, remains an unresolved inquiry. Abbreviations: HPA axis, hypothalamic-pituitary-adrenal axis; CRF, corticotropin-releasing factor; ACTH, adrenocorticotropic hormone; LPS, lipopolysaccharide; SCFAs, short-chain fatty acids; BBB, blood-brain barrier.

Stress has the potential to trigger seizures, and the HPA axis is the primary regulatory system in the stress response (Cano-López and González-Bono, 2019). Different hormones have distinct effects on seizures: glucocorticoid levels are elevated in epilepsy patients, while deoxycorticosterone, an anticonvulsant drug, exhibits beneficial effects; Corticotropin-releasing hormone (CRH) and corticosterone promote the signaling of excitatory neurotransmitters like glutamate, which can induce seizures (Baram and Schultz, 1991; Reddy and Rogawski, 2002; Werner and Coveñas, 2017). Recent research has revealed a correlation between gut microbiota and the HPA axis. For instance, structural and functional changes in the gut microbiota due to stressful conditions regulate the expression of genes associated with the colonic CRH pathway (Vodička et al., 2018) (see Figure 2). Nevertheless, further investigation is required to understand the mechanism connecting gut microbiota, the HPA axis, and epilepsy.

Congruent lines of evidence have proposed that imbalances in neurotransmitters have been strongly linked to the development of epilepsy, with epileptic foci often exhibiting decreased levels of inhibitory neurotransmitters, such as GABA, and elevated levels of excitatory neurotransmitters like glutamate, dopamine, and norepinephrine (Singh and Goel, 2016). For instance, the disrupted balance of GABA and glutamate in brain tissue has been observed in patients with epilepsy (Fisher et al., 2017b). GABA is an inhibitory neurotransmitter widely distributed in the nervous system, and a decrease in GABA levels can lead to changes in neuronal cell permeability to synaptic interstitial chloride ions. This alteration can result in lower seizure thresholds and an increased susceptibility to epilepsy (Barrett et al., 2012; Peng et al., 2018). It has been found that various types of gut microbiome are capable of metabolizing and producing distinct neurotransmitters. For example, *Enterococcus* spp., *Streptococcus* spp., and *Escherichia coli* spp. can produce serotonin. *Lactobacillus* spp. and *Bifidobacterium* spp. are responsible for synthesizing GABA. *Escherichia coli* spp. and *Bacillus* spp., on the other hand, produce noradrenaline and dopamine (Tsavkelova et al., 2000; Özoğul, 2004; Shishov et al., 2009; Barrett et al., 2012; Özoğul et al., 2012; Strandwitz, 2018) (see Figure 2). Recent studies have shown the relative abundance of intestinal *Ruminococcus* and *Coprococcus* was positively correlated with brain tissue glutamate and glutamine levels (Sun et al., 2016; Zhang et al., 2021; Binh Tran et al., 2023). The gut microbiota, specifically the presence of beneficial bacteria such as *A. mucinophilia* and *Parabacteroides*, may provide protective benefits against seizures by modulating brain neurotransmitter levels, including GABA and glutamate in the hippocampus. Bacterial dysbiosis may alter GABA levels, leading to an exacerbation of seizures (Galland, 2014; McDonald et al., 2018). In addition, Barrett et al. investigated that *Bifidobacterium* spp., an important probiotic in the gut, not only stimulates inflammatory responses and boosts the immune system’s defenses against external pathogens but also secretes GABA that may traverse the blood-brain barrier (BBB) and enter the CNS. Notably, when mice were colonized with Ackermannia and Paramycetes in their gastrointestinal tracts, it led to reduced levels of excitatory amino acids (glutamate) in both the serum and intestines, along with increased expression of GABA in the hippocampus. This colonization exerted anti-seizure effects (Olson et al., 2018). However, current research has yet to confirm the direct entry of neurotransmitters produced by gut microbiota into the brain across the BBB and their role in shaping brain structure and function.

Previous studies have demonstrated that approximately 90% of 5-HT is produced and distributed in intestinal enterochromaffin cells (ECs), but the intestinal microbiota can play a role in its production and secretion process (Yano et al., 2015; Lu et al., 2021). The commensal microbiota has the ability to directly utilize luminal tryptophan to synthesize serotonin. Various bacteria including *Lactococcus*, *Lactobacillus*, *Streptococcus*, *Escherichia coli*, and *Klebsiella* have been found to produce serotonin by expressing tryptophan synthetase. Specifically, strains such as *Lactococcus lactis subsp. Cremoris (MG 1363)*, *Lactococcus lactis subsp. Lactis (IL 1403)*, *Lactobacillus plantarum (FI8595)*, *Streptococcus thermophilus (NCFB2392)*, *Eschericchia coli K-12*, *Morganella morganii (NCIMB, 10466)*, *Klebisella pneumoniae (NCIMB, 673)*, and *Hafnia alvei (NCIMB, 11999)* have been identified as capable of serotonin production. Furthermore, it has been observed that commensal bacteria, particularly spore-forming bacteria found in the mouse and human microbiota, can enhance serotonin biosynthesis in colonic ECs through a metabolite or cell component-dependent mechanism (Williams and Chryssanthopoulos, 1997; Sudo et al., 2004; O'Mahony et al., 2015; Yano et al., 2015; Clark and Mach, 2016; Eisenstein, 2016; Mandic et al., 2019). For instance, specific microbiota within the intestines of mice have been found to promote the production of 5-HT by regulating the rate-limiting enzyme responsible for its synthesis, tryptophan hydroxylase 1 (TPH1) (Yano et al., 2015). Depletion of 5-HT induced by reserpine reduces the threshold for seizures triggered by electrical stimulation in rats, thereby increasing their susceptibility to seizures (Wenger et al., 1973). Therefore, it is reasonable to hypothesize that the intestinal microbiota modulates the production and release of 5-HT from endocrine cells, subsequently affecting the electrical activity of the intestinal vagus nerve and the immune-inflammatory response. This modulation, in turn, leads to an increased susceptibility to epilepsy. Besides, studies have revealed a reduction in brain tissue N-acetylaspartate (NAA) levels in both epileptic patients and animal models of epilepsy (Ferrier et al., 2000; Gomes et al., 2007).

Specific intestinal microbiota, such as Firmicutes and Bacteroidetes, have the ability to ferment and break down insoluble dietary fiber, leading to the production of important metabolites known as short-chain fatty acids (SCFAs), including acetate, propionate, and butyrate (den Besten et al., 2013) (see Figure 2). These SCFAs play a significant role in promoting the maturation of microglia, and changes in microglial function and BBB permeability have been linked to seizure susceptibility (De Caro et al., 2019a). It is worth noting that in this context, patients with drug-resistant epilepsy exhibited significantly different levels of Firmicutes and Bacteroidetes phylum compared to those with drug-sensitive epilepsy and healthy controls, as demonstrated in studies by Xie et al. (2017), Peng et al. (2018), Lindefeldt et al. (2019), Lee et al. (2020), Şafak et al. (2020), and Lee et al. (2021). A rat model of post-traumatic epilepsy caused by lateral fluid percussion injury showed that the trauma itself did not result in significant alterations in the gut microbiota. However, the risk of developing post-traumatic seizure was strongly correlated with the disruption of gut microbiota and a decrease in intestinal SCFAs content prior to the traumatic event (Medel-Matus et al., 2022). In an absence seizure model using WAG/Rij rats, it was observed that brain tissue levels of SCFAs were significantly reduced, whereas the supplementation of butyrate effectively controlled their absence seizures. This can be attributed to the ability of butyrate to enhance mitochondrial function, protect brain tissue from oxidative stress, and prevent neuronal apoptosis, ultimately increasing the seizure threshold and reducing seizure intensity (Li et al., 2021a). As such, propionate supplementation has been shown to attenuate mitochondrial damage, hippocampal apoptosis, and neurological deficits, leading to a decrease in seizure intensity and an increase in seizure latency (Cheng et al., 2019). Furthermore, the KD has been utilized for nearly a century as a treatment for drug-resistant epilepsy in both children and adults (Xie et al., 2017; Olson et al., 2018; Zhang et al., 2018; Lindefeldt et al., 2019; Eor et al., 2021; Gong et al., 2021; Miljanovic and Potschka, 2021; Shearer et al., 2023). A high consumption of cruciferous and leafy vegetables, berries, and nuts on the KD has been linked to a positive impact on the profile of SCFAs produced by the gut microbiome (Gudan et al., 2022).

## 5 The role of gut microbiota-mediated immune pathways in epilepsy

### 5.1 Gut microbiota in the immune system

The gut microbiota plays a crucial role in maintaining the balance of the host immune system. Research conducted on mouse models of multiple sclerosis and stroke has highlighted the involvement of gut microbes in the regulation of autoimmunity, particularly impacting the development and function of CNS-resident immune cells, such as microglia M1 (Ochoa-Reparaz et al., 2010; Wang et al., 2014b; Benakis et al., 2016). For instance, when adult SPF mice were treated with antibiotics to remove bacteria, microglia reverted to an immature state. However, subsequent restoration to a normal state was possible through allogeneic gut microbiota transplantation, demonstrating the intimate connection between gut microbial signaling and the maintenance of a mature microglial state (Erny et al., 2015). Plus, disruptions in T cell subset homeostasis, including Th1, Th2, Th17, and Treg, due to intestinal malnutrition, can contribute to the development of autoimmune and inflammatory disorders (Lee and Kim, 2017).

Th1 cells, which secrete pro-inflammatory cytokines like IL-2, IL-12, TNF-alpha, and IFN-γ, play a critical role in promoting cellular immune responses (Luckheeram et al., 2012). Recent research has shown that alterations in the gut microbiota composition leads to the accumulation of specific amino acids, such as phenylalanine and isoleucine, which in turn promote the differentiation and proliferation of Th1 cells. Consequently, Th1 immune cells infiltrate the brain and engage in local communication with M1 microglia, causing a shift towards a pro-inflammatory state. This cascade ultimately contributes to neuroinflammation and cognitive dysfunction associated with conditions like AD (Wang et al., 2019).

IL-17, which is produced by Th17 and γδT cells during acute infections, has been shown to contribute to the pro-inflammatory response. Interestingly, in stroke models, there is an enrichment of γδT cells in the gut, which are then transported to the delicate membranes of the brain. This suggests a potential link between gut inflammation and the onset of stroke (Weaver et al., 2007). On the other hand, Treg cells play a crucial role in maintaining immune homeostasis by secreting the anti-inflammatory cytokine IL-10, which helps to suppress excessive immune responses (Zhu et al., 2010). Animal models of stroke have demonstrated that the deletion of Treg cells leads to a significant increase in the activation of resident and infiltrating inflammatory cells, including microglia and T cells. This heightened activation consequently attenuates the post-ischemic inflammatory response (Liesz et al., 2009). Moreover, Treg cells have been found to inhibit the differentiation of Th17 cells and the proliferation of γδT cells in the gut, thereby contributing to the maintenance of an anti-inflammatory environment (Huber et al., 2011).

Fecal calprotectin, a protein involved in intestinal inflammation, has emerged as a potential regulator of the inflammatory response in neurodegenerative diseases like AD and PD. A study showed that fecal calprotectin levels were elevated in nearly 70% of AD patients, suggesting its role in promoting neuroinflammation (Leblhuber et al., 2015). Similarly, elevated levels of fecal calreticulin in PD patients led to changes in the integrity of the intestinal epithelial barrier and the immune system (Mulak et al., 2017). Calreticulin is a calcium-binding protein consisting of the S100A8 and S100A9 heterodimer, which make up a significant proportion of the soluble protein content of neutrophils. Both S100A8 and S100A9 are capable of producing amyloid proteins and can form associations with other amyloids like β-amyloid (Aβ) and alpha-synuclein (α-syn), as well as amyloid oligomers and protofibrils (Wang et al., 2014a; Walsham and Sherwood, 2016). Macrophages and microglia secrete S100A9 during amyloid plaque formation, leading to its expression in neuronal cells. These effector molecules interact with Toll-like Receptor 4 (TLR4) and Receptor for Advanced Glycation End Products (RAGE) pathways, further activating microglia. The increased levels of calreticulin in cerebrospinal fluid (CSF) and the brain of AD patients may facilitate its co-aggregation with Aβ and amyloid formation (Wang et al., 2014a).

Among the intestinal microbiota amyloids, the curli protein (curli) produced by *Escherichia coli* is currently the most extensively studied. It shares similarities in primary and tertiary structures with CNS amyloids and forms biofilms with bacterial cells, providing resistance against physical and immunological factors (Cherny et al., 2005; Zhao et al., 2015). Exposure to intestinal bacterial amyloid can activate the immune system, thereby enhancing the response to endogenous amyloid production in the brain (Friedland and Chapman, 2017). It has been demonstrated that exposure of rats to bacteria capable of producing frizzled protein leads to increased α-syn deposition in neurons, as well as higher numbers of microglia and astrocytes in both the gut and brain. This exposure also results in elevated expression of Toll-like Receptor 2 (TLR2), interleukin-6 (II-6), and tumor necrosis factor (TNF) in the brain (Chen et al., 2016).

### 5.2 Involvement of the gut microbiota-mediated immune response in epilepsy pathology

A cohort study conducted in northern Denmark, encompassing children born between 1998 and 2008, revealed a significant link between maternal infection during pregnancy and an increased risk of epilepsy in their offspring (Nørgaard et al., 2012). It is worth noting that this outcome is not specific to any particular pathogens or drugs but rather can be attributed to common processes associated with infections. Corroborating this finding, another cohort study examining Danish births from 1982 to 2012 reported a noteworthy 78% surge in the prevalence of epilepsy among children and young adults who had been hospitalized for an infection (Ahlers et al., 2019). The infants affected by *group B streptococcus* (GBS) infection, a leading cause of neonatal infections, faced a significantly elevated risk of developing epilepsy or other neurological disorders later in life (Yeo et al., 2019). The impact of infectious diseases on epilepsy is further amplified by epidemiological investigations from Norway, which demonstrated a significant rise in the incidence of infection-associated febrile seizures following the 2009 influenza A (H1N1) outbreak (Bakken et al., 2015). Furthermore, an association has been established between human herpesvirus type 6 (HHV-6) and medial temporal lobe sclerosis, a common cause of refractory epilepsy. For instance, infected individuals with medial temporal lobe sclerosis experience more frequent seizures and exhibit higher viral DNA loads indicative of HHV-6 involvement in the development of this condition through the induction of an aberrant immune-inflammatory response (Kawamura et al., 2015; Leibovitch and Jacobson, 2015). These findings collectively emphasize the critical role that infections (or immune-inflammatory response), both during pregnancy and throughout early life, play in shaping the risk and development of epilepsy.

**FIGURE 3:**
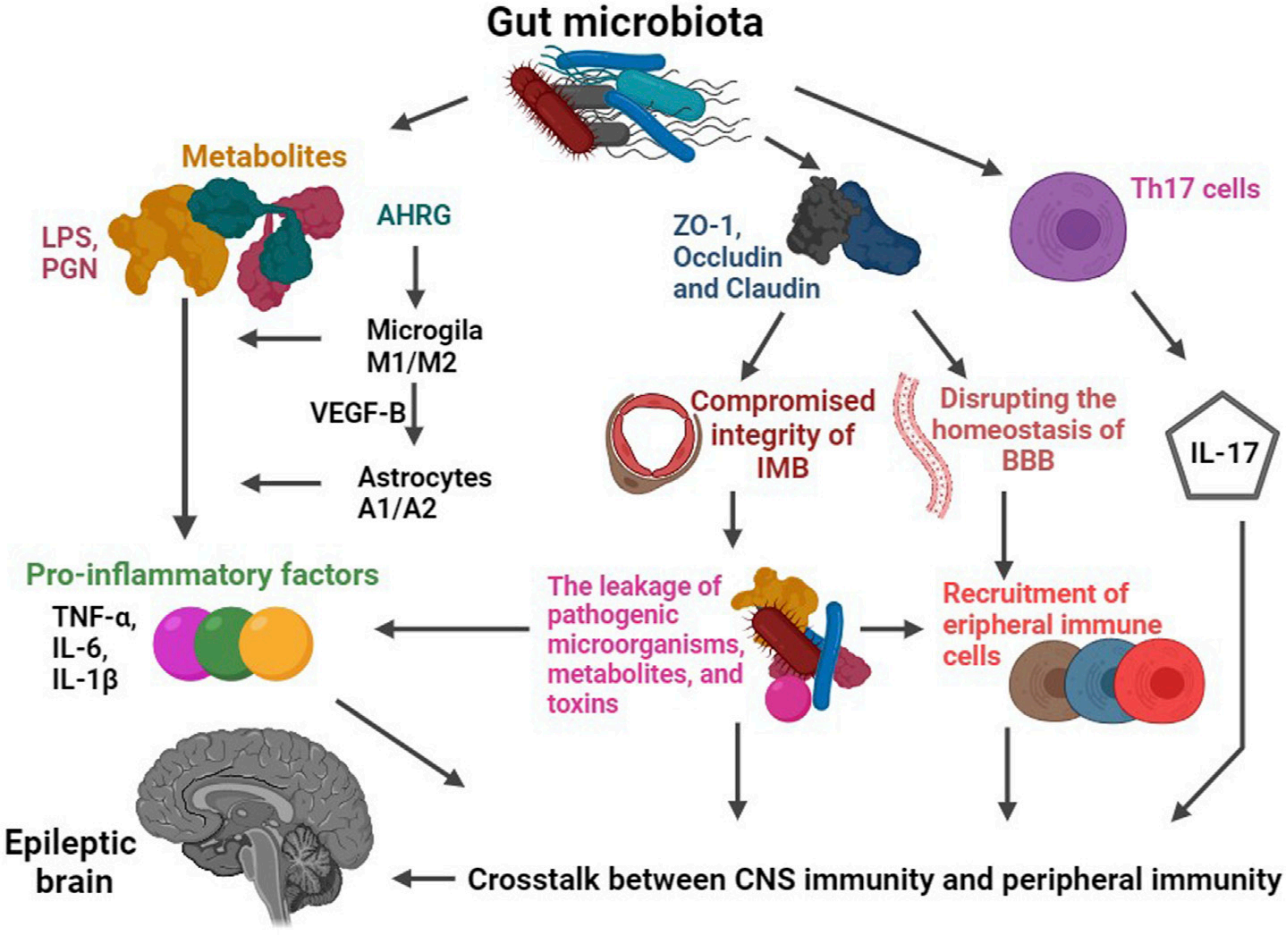
Schematic diagram of the role of gut microbiota-mediated immune pathways in epilepsy. The gut microbiota plays a crucial regulatory role in the expression of key proteins responsible for maintaining the integrity of the intestinal mucosal barrier (IMB) and blood-brain barrier (BBB), such as Occludin and Claudin. When the permeability of these barriers is increased, it paves the way for the escape of pathogenic microorganisms, metabolites (such as LPS and PGN), and toxins from the intestinal tract. Consequently, compromised barriers allow for a significant influx of immune cells (including peripheral monocytes or macrophages, T-cells) and inflammatory factors (such as TNF-A and IL-1b) to enter the CNS. These immune cells and inflammatory factors then interact with central immune components like microglia and astrocytes, ultimately triggering seizures. Furthermore, specific intestinal microbiota, such as those belonging to the Bacteroidetes phylum, can modulate the secretion of IL-17. These gut bacteria have the ability to influence the activation and differentiation of Th17 cells, leading to alterations in the secretion of IL-17. Abbreviations: LPS, lipopolysaccharide; PGN, peptidoglycan; AHRG, aryl hydrocarbon receptor agonist; VEGF-B, vascular endothelial growth factor-B; IMB, intestinal mucosal barrier; BBB, blood-brain barrier.

Evidence from animal models further supports the notion that infections heighten the risk of developing epilepsy by engaging immunoinflammatory pathways, involving factors such as TNF-α, monocyte chemoattractant protein-1 (MCP-1), and others (Lv et al., 2014; Cusick et al., 2017; Patel et al., 2017; Sewal et al., 2017; Medel-Matus et al., 2018). For instance, studies utilizing rodent models have demonstrated that the injection of lipopolysaccharide, a component found in bacterial cell walls, triggers an upsurge in pro-inflammatory factors like TNF-α, IL-6, and IL-1β within brain tissue. This inflammatory response consequently lowers the threshold for seizures induced by chemical (PTZ) and electrical stimulation (Kołosowska et al., 2014; Russo et al., 2014; Leo et al., 2020; Hu et al., 2022) (see Figure 3). Intracerebral infection of C57BL/6J mice with the Daniels (DA) strain of Theiler’s murine encephalomyelitis virus (TMEV) leads to approximately 50% of the mice exhibiting acute behavioral seizures. The primary pathophysiological mechanism involves elevated levels of inflammatory factors in the hippocampus and limbic system, particularly IL-6 and TNF-α (Cusick et al., 2017). Remarkably, when IL-6 and TNF receptor 1 (TNFR1) and TNF receptor 2 (TNFR2) knockout mice were injected with TMEV, a significant reduction in seizures was observed, highlighting the key role of these inflammatory factors in the epileptic process (Cusick et al., 2017).

Th17 cells, identified as CD4^+^ T cell subset, hold a pivotal role in orchestrating the adaptive immune response (Weaver et al., 2007; Chow and Mazmanian, 2009; Huber et al., 2011; Lee and Kim, 2017). Their production of IL-17 can be modulated by specific intestinal flora, such as those belonging to the Bacteroidetes phylum. These gut bacteria can influence the activation and differentiation of Th17 cells, consequently altering the secretion of IL-17 (Ivanov et al., 2008) (see Figure 3). Notably, patients suffering from epilepsy exhibit markedly elevated levels of IL-17 in both cerebrospinal fluid and peripheral blood. In line with this, alterations in the expression of IL-17 showcase a positive correlation with both seizure frequency and severity. This intriguing link suggests that the gut flora may influence epilepsy susceptibility by intricately mediating the IL-17 pathway (Mao et al., 2013).

The pathogenesis of epilepsy is intertwined with the inflammatory pathways that are regulated by the brain-gut-microbiota axis (Gong et al., 2022b; Ding et al., 2022; Gernone et al., 2022; Mu et al., 2022). For instance, the composition of the gut flora may influence the immune function and modulate the susceptibility of hosts to seizures (Wu et al., 2016; Li et al., 2021b) (see Figure 3). Specifically, gut microbiota metabolizes dietary tryptophan into an aryl hydrocarbon receptor agonist. This agonist, in turn, interacts with microglia receptors, promoting their migration, apoptosis, and phagocytosis, ultimately leading to increased release of inflammatory factors (Ochoa-Reparaz et al., 2010; Wang et al., 2014a; Benakis et al., 2016; Rothhammer et al., 2018). Additionally, the activation of microglia further upregulates the expression of transforming growth factor-alpha (TGF-α) and vascular endothelial growth factor-B (VEGF-B) in astrocytes. As a consequence, an inflammatory response is triggered within astrocytes (Rothhammer et al., 2018). The crosstalk between astrocytes and microglia can potentiate an enhanced inflammatory response and disrupt the homeostasis of the BBB, facilitating the entry of peripheral blood immune cells and inflammatory factors into the CNS. This chronic inflammation, in turn, significantly elevates the risk of epilepsy (Rothhammer et al., 2018). Experimental evidence supports the role of gut flora in maintaining microglial immune function (Erny et al., 2015; Rothhammer et al., 2016). For instance, observations in mice reared in a sterile environment or treated with antibiotics to suppress gut flora reveal that microglia in these animals exhibit morphological, functional, differentiation, and activation defects, leading to innate immunodeficiencies against pathogens. Since there is no evidence to support that this pathological elimination of microbiomes does not affect the absorption of nutrients, exclusion of toxins, migration of cells, or other effects at a gut level, which could potentially have a multistep downstream effect on microglia. Therefore, it is reasonable to hypothesize that microglial changes are primarily an indirect result of changes in the microbiota. However, upon recolonization of the gastrointestinal tract, the immune function of these microglia is restored, underscoring the criticality of gut microbiota in regulating microglial function (Rothhammer et al., 2016). Moreover, abnormalities in the function of peripheral immune cells have been implicated in the development of epilepsy. T-lymphocytes and monocytes, upon migration to the CNS, can differentiate into macrophages and invade brain tissue, ultimately inducing seizures (Erny et al., 2015). Lin et al. (2021) demonstrated an increase in gut *Klebsiella pneumoniae* in patients with epilepsy. Subsequent animal studies have shed light on the impact of *Klebsiella pneumoniae* in the intestinal tract on seizure susceptibility and the activation of microglial cells to release inflammatory factors (Lin et al., 2021). This discovery highlights the intricate interplay between neuroinflammation and epilepsy pathogenesis. All told, the deep involvement of gut flora in both peripheral and neuroinflammation strongly suggests that gut flora-mediated neuroinflammation may play an important role in epileptogenesis that must not be overlooked (see Figure 3).

Intestinal microbiota exerts a regulatory role on the expression of key proteins involved in the integrity of the intestinal mucosal barrier, such as Occludin and Claudin. Animal studies have demonstrated a significant increase in BBB permeability in mice reared in a germ-free environment or treated with antibiotics to inhibit gut microbiota (Amasheh et al., 2011; Braniste et al., 2014). Dysbiosis of gut microbiota may downregulate the expression of Occludin and Claudin, leading to compromised integrity of the intestinal mucosal and BBB (see Figure 3). Consequently, this increased permeability facilitates the leakage of pathogenic microorganisms, metabolites, and toxins from the intestinal tract, and then the compromised barriers allow a large influx of immune cells and inflammatory factors to enter the CNS, ultimately triggering seizures (Rahman et al., 2018; Vezzani et al., 2019; Welcome, 2019). For instance, studies have found reduced expression levels of occludin and ZO-1, crucial components of the tight junctions in the BBB, in patients with drug-resistant temporal lobe epilepsy (Castañeda-Cabral et al., 2020). Besides that, the gut harbors a vast population of bacteria, many of which possess cell wall components, such as peptidoglycan (PGN) (Clarke et al., 2010). PGN and its fragments possess strong proinflammatory properties, signaling through Toll-like receptors (TLR), NOD-like receptors (NLR), and specialized PGN recognition proteins (such as PGLYRP1-4) (Laman et al., 2020). Alterations in the permeability of the intestinal mucosal barrier allow flora-associated metabolites, including PGN, to cross into the CNS. As a consequence, chronic inflammation is induced within the CNS, further contributing to the pathogenesis of epilepsy (Gales and Prayson, 2017). The collective findings from the aforementioned studies strongly implicate the involvement of intestinal flora in the regulation of epilepsy susceptibility through its influence on barrier permeability and immune-inflammatory responses. Although these studies provide valuable insights, it is important to note that only a limited number have investigated the intricate relationship between gut microbiota, immune-inflammatory responses, and epilepsy. Therefore, further well-designed studies are imperative to corroborate and expand upon these findings, in order to fully elucidate the role of the brain-gut-microbiota axis in the pathogenesis of epilepsy.

Significantly, epidemiological studies have established a clear association between inflammatory gastrointestinal diseases (GI) and the incidence of epilepsy. For instance, patients with epilepsy have been found to have up to eight times higher incidence of peptic ulcers compared to the general population (Keezer et al., 2016). Furthermore, a perforated peptic ulcer can trigger or complicate a generalized tonic-clonic seizure (Magon, 2010). Huang et al. (2010) demonstrated that even mild gastroenteritis could precede the development of benign infantile convulsions. Neurological complications are also observed in a considerable percentage of patients with inflammatory bowel disease (IBD), ranging from 0.25% to 47.50%. In severe cases of IBD, seizures of all types, including status epilepticus, can occur during the clinical course (Ferro and Oliveira Santos, 2021). Based on this evidence, it is plausible to hypothesize that some cases of epilepsy may be caused by autoimmune disorders that have manifestations in both the gut and the brain, with the direction of causality attributed to an extraneous factor (i.e., neither epilepsy nor the gut microbiome). Consider the example of Celiac disease, a well-documented systemic autoimmune condition characterized by gluten-triggered autoimmune intestinal villous atrophy, malabsorption, and a range of systemic and gastrointestinal symptoms. Approximately 10% of individuals with celiac disease experience neurological complications, including seizures. Conversely, about 0.78%–9.10% of epilepsy patients develop celiac disease (Djurić et al., 2012; Işıkay and Kocamaz, 2014). The precise mechanism behind these neurological manifestations remains poorly understood, likely linked to immune mechanisms. This hypothesis is supported by the presence of anti-Purkinje cells and anti-ganglioside antibodies in individuals with celiac disease who develop neurological symptoms (Pratesi et al., 2003). Moreover, drug-resistant epilepsy is more prevalent in children with celiac disease as a comorbidity. Remarkably, adherence to a gluten-free diet has led to the resolution of epilepsy in many celiac disease patients. Furthermore, maintaining a gluten-free diet alongside appropriate antiseizure medications has shown to reduce seizure frequency and severity in individuals with celiac disease and drug-resistant epilepsy (Swartwood et al., 2021). In these scenarios, it is plausible that a reduction in the inflammatory state, possibly through dietary changes, may significantly impact both gut microbiota and the severity of epilepsy. Nevertheless, these hypotheses necessitate further research to validate their potential implications.

## 6 Gut microbiota in epilepsy treatment

### 6.1 The KD is a highly promising dietary intervention strategy with extraordinary potential for seizure control

Dr. Russell Wilder in the 1920s noted that seizures were halted during states of absolute fasting (Wheless, 2008; Winesett et al., 2015). With this observation, Dr. Wilder formulated a diet that emulated the effects of fasting by relying on a high-fat, moderate-protein, and low-carbohydrate composition. Specifically, the KD adheres to a calculated ratio of fat to the combined intake of proteins and carbohydrates, typically ranging between 2:1 and 4:1. To illustrate, a 4:1 ratio involves consuming four servings of fat for every one serving of proteins and carbohydrates. Therefore, 90% of the body’s required calories are derived from fat, while adequate protein is included to prevent the utilization of lean body mass. By metabolizing fat into ketone bodies, the brain is provided with an alternative fuel source to glucose, which is typically utilized when carbohydrates are consumed in excess.

Over the years, the dietary regimen developed by Dr. Wilder remains the foundation of KD therapy used today. Simultaneously, less strict variations of the original diet have also been introduced, including the Modified Atkins diet (MAD) and low glycemic index treatment (LGIT) (Kossoff and Doward, 2008; Pfeifer et al., 2008). Further, extensive exploration into the potential applications of the KD has expanded its use beyond epilepsy to other neurological disorders such as Alzheimer’s disease and Parkinson’s disease, among others. In recent years, the diet has even been investigated as a potential therapeutic intervention for non-neurological diseases. However, its foremost role remains as an effective treatment for epilepsy (Winesett et al., 2015; Xie et al., 2017; Olson et al., 2018; Zhang et al., 2018; Lindefeldt et al., 2019; Li et al., 2021b; Eor et al., 2021; Gong et al., 2021; Miljanovic and Potschka, 2021; Gudan et al., 2022; Shearer et al., 2023). In the pediatric age group, the KD has demonstrated significant benefits. In one study, it resulted in a 50% reduction in seizure frequency for at least 56% of patients, a 90% reduction in seizures for 32% of patients, and complete cessation of seizures in 16% of patients (Lefevre and Aronson, 2000). In a 2008 clinical trial conducted in England, children were randomly assigned either to receive the KD after a 1-month delay (treatment arm) or to undergo a 4-month delay (control arm) with no changes to their anticonvulsant medications. Five out of 54 patients in the treatment arm experienced a remarkable 90% reduction in seizures, while none of the patients in the control arm achieved such a response at the 4-month mark. Moreover, a greater than 50% decrease in seizures was observed in 38% of the patients in the treatment arm, compared to only 6% in the control arm. It is important to note that these findings were particularly significant considering the patients in the study were classified as quite refractory, having previously failed multiple antiseizure drugs (Neal et al., 2008).

Another study by Guzel et al. examined the effects of a 1-year KD intervention on 389 patients with drug-resistant epilepsy. The results revealed a significant improvement in the anti-seizure effect of the KD, with sustained efficacy over time. At 1, 3, 6, and 12 months, 65.8%, 74.7%, 70.6%, and 83.1% of the patients, respectively, demonstrated a positive response to the treatment (Guzel et al., 2019). The broad efficacy of the KD in treating various seizure types and childhood epilepsy syndromes, including symptomatic generalized, partial, and genetic epilepsies, is worth noting (Hartman, 2008; Hara et al., 2018). In fact, certain subgroups of epilepsy syndromes exhibit a particularly favorable response to the KD compared to available pharmacological treatments. One such remarkable subgroup is patients with glucose transporter (Glut-1) deficiency syndrome, where the efficacy of the KD may exceed 80% (Hartman, 2008; Leen et al., 2010). Recent research has delved into the effectiveness of combining the MAD with standard drug therapy (SDT) in the treatment of drug-resistant epilepsy. The findings of the study revealed that the combination of MAD and SDT was superior to SDT alone in reducing seizure frequency and improving overall outcomes, at the 6-month mark in adolescents and adults with non-surgical drug-resistant epilepsy (Manral et al., 2023). Moreover, the KD has demonstrated benefits beyond seizure control. It has been found to have a positive impact on the cognitive, emotional, verbal, and intellectual development of children (Zhu et al., 2016). However, KD or dietary treatment for seizures has thus far only shown effectiveness in pediatric populations. There is currently a lack of concrete evidence to support its benefits or effectiveness in adult populations. As a result, all the KD-related patients we discuss below are from studies focused on pediatric epilepsy.

### 6.2 Anti-seizure effects of the KD mediated by the gut microbiota

**FIGURE 4:**
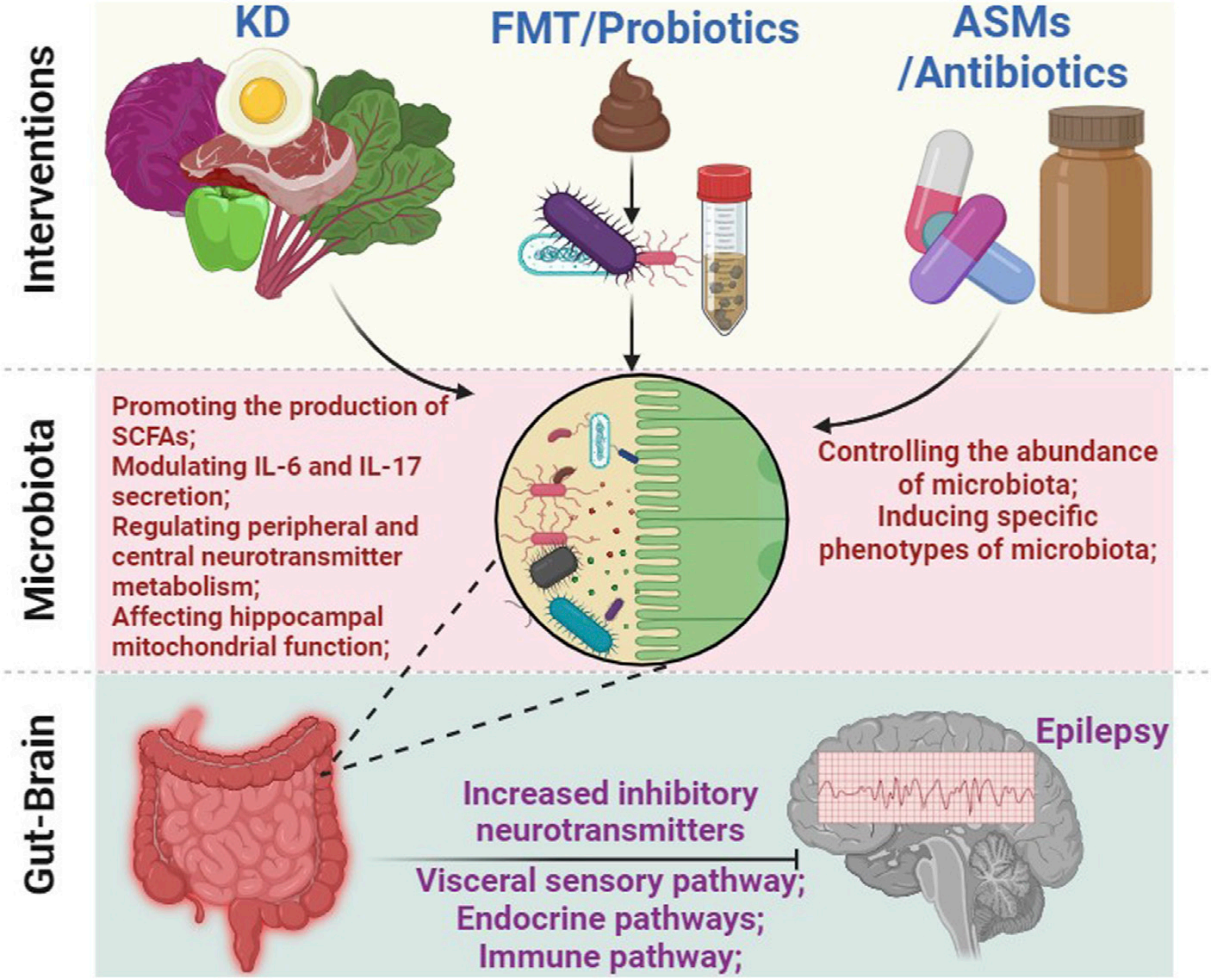
The intricate connection between gut microbiota and the treatment of epilepsy. Various approaches, including KD, FMT, probiotics, antibiotics, and ASMs, can affect the composition and function of gut microbiota. This, in turn, triggers a cascade of effects leading to alterations in inflammation response, peripheral and central metabolites (such as short-chain fatty acids, 5-HT, and GABA, among others), as well as the activity of the ENS, ultimately modulating brain function and effectively controlling seizures. Abbreviations: KD, ketogenic diet; FMT, fecal microbiota transplantation; ASMs, anti-seizure medications; ENS, enteric nervous system; SCFAs, short-chain fatty acids.

A growing body of evidence suggests that the gut microbiota may play a significant role in the therapeutic effects of the KD for epilepsy (Olson et al., 2018; Spinelli and Blackford, 2018; Dahlin and Prast-Nielsen, 2019; Lindefeldt et al., 2019; Pittman, 2020; Ding et al., 2021; Yue et al., 2022; Özcan et al., 2022; García-Belenguer et al., 2023; Kundu et al., 2023; Shearer et al., 2023) (see Figure 4). A cross-sectional study conducted with 12 children suffering from drug-refractory epilepsy examined the impact of a 6-month KD treatment. The results were remarkable, with one patient experiencing complete cessation of seizures, three patients achieving a staggering 90%–99% reduction in seizure frequency, and five out of twelve children achieving a reduction of over 50% in seizure frequency. Cognitive and motor function improvements were observed in ten out of twelve children after 6 months on the KD. Importantly, a comparison of fecal samples taken before and after the 6-month KD intervention showcased that there was a notable decrease in the abundance of *Bifidobacterium*, *Akkermansia*, *Enterococcaceae*, and *Actinomyces*, along with a significant increase in the abundance of *Subdoligranulum*, *Dialister*, *Alloprevotella*, and *Bifidobacterium* (Gong et al., 2021). Another study conducted with 20 children suffering from refractory epilepsy who followed a KD for 6 months also yielded similar results. Two patients became completely seizure-free, three experienced a seizure reduction of 90% or more, five achieved a reduction between 50%–89%, and ten saw a reduction of less than 50%. Remarkably, all ten responders showed an improvement in their electroencephalogram (EEG) readings. In addition, analysis of fecal microbial profiles revealed a lower alpha diversity after KD therapy, along with significantly decreased abundance of Firmicutes and increased levels of Bacteroidetes (Zhang et al., 2018).

A significant increase in the abundance of the Bacteroidetes phylum within the intestinal flora was observed following KD treatment. This shift in microbial composition has been associated with the modulation of interleukin 6 and interleukin 17 secretion in dendritic cells, as well as promoting the production of SCFAs. These alterations play a key role in the reduction of seizure severity (Olson et al., 2018; Lindefeldt et al., 2019). Animal studies have also provided evidence supporting the efficacy of the KD in increasing seizure thresholds. In mice with a 6-Hz-induced refractory seizure model, the KD demonstrated the ability to raise seizure thresholds (Samala et al., 2008; Hartman et al., 2010; Olson et al., 2018). The KD also induced rapid and significant changes in the composition of the mouse gut microbiota. For instance, it led to an increase in the relative abundance of *Akkermansia muciniphila* and *Parabacteroides* (Samala et al., 2008; Hartman et al., 2010; Olson et al., 2018). To delve deeper into the relationship between the gut microbiota and the protective effects of the KD, Olson et al. conducted experiments involving germ-free feeding or antibiotic treatment (consisting of ampicillin, vancomycin, neomycin, and metronidazole) followed by 6 Hz electrical stimulation to induce epilepsy modeling. The results revealed that the protective effects of the KD were attenuated in germ-free fed and antibiotic-treated mice. However, when germ-free fed mice received the intestinal microbiota from normal mice, the seizure-protective efficacy was restored to that observed in normal mice receiving the KD. These findings strongly suggest that the presence of a healthy gut microbiota is necessary for the KD to exert its seizure-protective effects (Olson et al., 2018). Further investigations have indicated co-administration of *Akkermansia muciniphila* and *Parabacteroides* to the gastrointestinal tracts of germ-free or antibiotic-treated mice resulted in a notable increase in seizure thresholds upon 6 Hz electrical stimulation, supporting that the enriched intestinal flora associated with the KD possess anti-seizure properties (Frost et al., 2014; Vuong et al., 2017; Olson et al., 2018). Interestingly, colonization with only *Akkermansia muciniphila* or *Parabacteroides* alone did not restore the protective effect of the KD. This indicates that the presence of both bacteria is necessary for the anti-seizure effect of the KD (Olson et al., 2018). The metabolomic studies have suggested that administration of the KD resulted in decreased peripheral blood γ-D-glutamine levels, which was associated with an increase in the hippocampal GABA/glutamate ratio in mice, suggesting that the gut flora may modulate peripheral metabolites and central neurotransmitter metabolism, ultimately regulating seizure susceptibility (Frost et al., 2014; Vuong et al., 2017; Olson et al., 2018). The transplantation of KD-associated intestinal flora (*Akkermansia muciniphila* and *Parabacteroides*) into the gut of *Kcna1* ^*−/−*^ sudden epileptic death model mice demonstrated elevated seizure thresholds, reduced seizure frequency, and shortened seizure duration. Instead, inhibition of both bacteria with antibiotics led to an increase in spontaneous tonic-clonic seizures in mice. These results highlight the broad applicability of the anti-seizure effects of the intestinal flora to various seizure types and model mice (Fenoglio-Simeone et al., 2009; Olson et al., 2018).

Remarkably, the manipulation of intestinal flora in models of IS using a KD and antibiotics has been shown to impact hippocampal mitochondrial function and subsequently alter epilepsy susceptibility. This observation suggests that targeting mitochondrial function-related flora could hold promise as a potential therapeutic strategy for epilepsy (Mu et al., 2022b). The major metabolite of ginsenosides Rb1, Rb2, and Rc in the intestinal microbiota, Ginsenoside compound K (GCK), has been found to have beneficial effects in a rat model of PTZ-induced epilepsy. GCK increases the levels of GABA, ultimately leading to a reduction in the intensity and prolongation of seizure latency (Zeng et al., 2018).

The disparities observed between animals in a germ-free environment and control animals are indeed fascinating (Fenoglio-Simeone et al., 2009; Frost et al., 2014; Vuong et al., 2017; Olson et al., 2018). However, it is crucial to approach the physiological effects of a germ-free environment with caution, as it represents an extreme condition that profoundly impacts the health and wellbeing of animals. Hence, it is imperative to build on these studies by conducting a thorough evaluation of the significance of the findings and delving deeper into the causation that precedes the observed factors.

### 6.3 FMT, probiotics and epilepsy treatment

FMT is an increasingly promising strategy for reconstructing the gut microbiota and has shown efficacy in various disease, such as epilepsy, *Clostridium difficile* infection, IBD, constipation, and other related disorders (Hartman et al., 2010; Borody and Khoruts, 2011; Surawicz et al., 2013; Reinisch, 2017; Olson et al., 2018). In a notable case study, a patient with both Crohn’s disease and refractory epilepsy underwent fecal transplantation, resulting in remarkable improvements. Following three consecutive transplants, the Crohn’s disease activity index (CDAI) significantly decreased from 361 to 131. Also, the patient’s seizures were completely controlled without the need for anti-seizure drugs 20 months after the fecal transplantation (He et al., 2017). However, this case is unique because it potentially involves an autoimmune etiology of Crohn’s disease as well as epilepsy. In another study, the administration of a probiotic mixture consisting of *Lactobacillus acidophilus DSM32241*, *Lactobacillus plantarum DSM32244*, *Lactobacillus casei DSM32243*, *Lactobacillus helveticus DSM32242*, *Lactobacillus brevis DSM11988*, *Bifidobacterium lactis DSM32246*, *B. lactis DSM32247*, and *Streptococcus salivarius* subsp. *thermophilus DSM32245* in patients with drug-resistant epilepsy for four consecutive months yielded promising outcomes. Notably, 28.9% of the patients experienced a significant reduction of more than 50% in the frequency of seizures (Gómez-Eguílaz et al., 2018). Furthermore, the administration of probiotics, specifically *Saccharomyces boulardii* or *Lactobacillus casei*, to neonates within 24 h of birth has displayed promising effects in reducing the risk of seizures. This effect was observed regardless of whether the infants were infected with rotavirus or not. Conversely, rotavirus-infected infants who did not receive probiotics exhibited an increased risk of seizures (Yeom et al., 2019). One potential mechanism underlying the anti-seizure effect of *Saccharomyces boulardii* in rotavirus-infected infants involves the inhibition of rotavirus non-structural protein 4 (NSP4). NSP4 has been implicated in increasing reactive oxygen species (ROS) production, white matter damage, and immune-inflammatory responses. By inhibiting NSP4, *Saccharomyces boulardii* may mitigate these detrimental effects, thereby reducing the frequency and severity of seizures in these infants (Buccigrossi et al., 2014; Yeom et al., 2019) (see Figure 4).

Animal models have also provided evidence for the anti-seizure effects of probiotics (see Figure 4). Studies involving rat and mouse brain tissues have shown that the application of *Lactobacillus rhamnosus* and/or *Bifidobacterium longum* leads to increased expression of GABA and its receptor GABAR, resulting in a reduction in seizures (Bravo et al., 2011; Liang et al., 2017). Furthermore, the administration of probiotic supplements containing *Lactobacillus rhamnosus*, *Lactobacillus reuteri*, and *Bifidobacterium infantis* to PTZ-induced epilepsy model rat for 3 weeks demonstrated favorable outcomes. The intervention resulted in a decrease in epileptic activity levels, a significant reduction in seizure severity, and a partial improvement in spatial learning and memory in the epileptic rats. Importantly, the probiotic treatment also led to decreased concentrations of pro-inflammatory cytokines IL-6 and TNF-α, reduced levels of nitric oxide (NO) and malondialdehyde (MDA), increased total antioxidant capacity (TAC), and elevated levels of GABA in the brain tissue (Bagheri et al., 2019; Aygun et al., 2022a; Aygun et al., 2022b). Additional research utilizing animal models of epilepsy has corroborated the anti-seizure effects of probiotics. For instance, the supplementation of probiotics and NS (N-stearoyl-L-tyrosine) reduced population spike-long-term potentiation (PS-LTP) in kindled rats, thereby improving seizure-induced cognitive impairment (Gong et al., 2020). Other animal models have also demonstrated the potential anti-seizure effects of probiotics (Tahmasebi et al., 2020; Eor et al., 2021; Kilinc et al., 2021; Sabouri et al., 2021; Mu et al., 2022b; Wang et al., 2022; Ciltas et al., 2023).

### 6.4 Gut microbiota and anti-seizure medications

Anti-seizure medications (ASMs) are typically the first line of treatment for controlling seizures in patients with epilepsy. However, a significant portion of these patients, estimated to be around 30%–40%, do not respond to at least two ASMs. Drug-resistant epilepsy patients also tend to exhibit dysregulation in their gastrointestinal ecological system, highlighting the potential involvement of the gut microbiota in epilepsy treatment (Peng et al., 2018). Drug-resistant patients have been found to possess higher alpha diversity, indicating a greater number of unique microbial types compared to drug-sensitive patients. Furthermore, specific microbiota has been associated with drug response. For instance, drug-sensitive patients tend to have a greater relative abundance of *Bacteroides* and *Ruminococcus*, while members of the *Negativicute*s group are overrepresented in drug-resistant epilepsy patients (Peng et al., 2018; Lee et al., 2021). Further evidence supporting the role of the gut microbiota in drug-resistant epilepsy comes from both animal models and pediatric cohort studies. These studies have demonstrated that the microbiota-based modulation of the anticonvulsant properties of the KD has shown promise in improving seizure control in these patients (Olson et al., 2018; Lindefeldt et al., 2019) (see Figure 4).

Previous research has demonstrated that intestinal inflammation can reduce the effectiveness of VPA, while therapy with SCFAs can effectively reduce inflammation, thereby enhancing the efficacy of VPA treatment (De Caro et al., 2019b). In animal models of epilepsy, the administration of lithium, valproate, and aripiprazole has been found to significantly increase the richness and diversity of microbial species. At the phylum level, valproate treatment has been shown to induce an increase in Actinobacteria and Firmicutes, while simultaneously decreasing the abundance of Bacteroidetes. At the genus level, several species including *Clostridium*, *Peptoclostridium*, *Intestinibacter*, and *Christenellaceae* were found to be increased following treatment with lithium, valproate, and aripiprazole compared to the control rats (Cussotto et al., 2019). Additionally, valproate treatment in rat models has been shown to decrease the relative abundance of *S24-7 uncultbact* and increase the relative abundance of *Ruminococcaceae* uncultured. Interestingly, VPA was also found to decrease the levels of two important SCFAs, namely, propionate and butyrate, in the cecum, while increasing the levels of isovalerate (Cussotto et al., 2019). Notably, mice exposed to sodium valproate demonstrated an increased ratio of Bacteroidetes to Firmicutes when compared to control mice (Sgritta et al., 2019). In clinical studies, it has been observed that the ratio of the phylum Firmicutes to Bacteriodetes is significantly higher in patients with new-onset epilepsy after 3 months of VPA treatment (Gong et al., 2022a). These findings provide further support for the role of gut microbiota composition in epilepsy and suggest that alterations in microbial populations may be associated with the effects of VPA therapy.

The anti-seizure drug lamotrigine and its ammonium salt complexes exhibit strong antibacterial activity against Gram-positive bacteria such as *B. subtilis*, *S. aureus*, and *S. faecalis* (Qian et al., 2009). Clonazepam, another anti-seizure drug, is metabolized by intestinal flora, which can potentially increase the drug’s toxic effects (Zimmermann et al., 2019). Then carbamazepine has been found to induce specific phenotypes in gut microbiota (Watkins et al., 2017; Gong et al., 2022; Ilhan et al., 2022). In a study involving epilepsy patients, after 3 months of carbamazepine treatment, the frequency of seizures was reduced by more than 50%. The abundance of Actinobacteria at the phylum level, which was initially higher in epileptic patients compared to healthy controls, decreased significantly after treatment. At the genus level, the abundances of bacteria such as *Escherichia/Shigella*, *Streptococcus*, *Collinsella*, and *Megamonas* were significantly higher in epilepsy patients before treatment but decreased significantly after treatment (Gong et al., 2022).

ASMs typically possess a narrow therapeutic window and are associated with numerous adverse effects, particularly impacting the gastrointestinal tract. Between 10% and 30% of epilepsy patients experienced intolerable side effects from these medications, leading them to discontinue treatment, particularly when undergoing polytherapy (Wiebe et al., 2008). Many ASMs have a direct impact on the enteric nervous system, influencing gut motility, which is evident in the gastrointestinal adverse effects of these medications (Jahromi et al., 2011; Minton et al., 2011; Al-Beltagi, 2021; Al-Beltagi and Saeed, 2022; Nevitt et al., 2022). For example, carbamazepine can lead to a range of gastrointestinal adverse effects, including dry mouth, mouth sores, glossitis, loss of appetite, dysphagia, nausea, vomiting, heartburn, gastritis, stomach/abdominal pain, constipation, and diarrhea (Anttila and Valtonen, 1992). Similarly, ethosuximide can induce anorexia, nausea, vomiting, gastric pain, diarrhea, and gastric and intestinal atony with decreased peristaltic activity (Kristev et al., 1994; Zagorchev et al., 1998). Phenobarbital can result in sore throat, diarrhea, swelling of the tongue/throat, nausea, vomiting, constipation, dysphagia, and heartburn (Jahromi et al., 2011; Al-Beltagi and Saeed, 2022). Phenytoin can cause changes in taste sensation, gingival overgrowth, sore throat, mouth ulcers, diarrhea, nausea, vomiting, constipation, dysphagia, heartburn, and reduced gastrointestinal absorption of calcium (Bell et al., 1979). Valproate also leads to gastrointestinal adverse effects, including diarrhea, nausea, vomiting, constipation, dysphagia, and gastritis with heartburn (Abdullah and Mousheer, 2020). This influence on the gut may significantly contribute to alterations in the gut microbiome. However, despite our comprehensive review of the existing research literature, we have found a lack of studies specifically examining the microbiota alterations induced by the gastrointestinal adverse effects of ASMs.

Carbamazepine, lamotrigine, and topiramate have been found to inhibit the growth of more than ten strains of bacteria when combined with the syrup excipient propyl-paraben. Ilhan et al. (2022) studied the effects of sweeteners and benzoates commonly found in ASM syrup. They found that the impact of these chemicals was concentration-dependent, with parabens and sweeteners showing increased toxicity or proliferation with higher concentrations. *Bifidobacterium species* were negatively affected by high concentrations of propyl-paraben, while methyl-paraben did not have the same effect. These results align with previous research by Crovetto et al., who reported a greater toxic effect of propyl- and butyl-parabens compared to methyl-paraben on *E. coli* and *Staphylococcus aureus* under aerobic conditions (Cussotto et al., 2019). Interestingly, the presence of various artificial sweeteners in ASMs formulations stimulated the growth of certain gut bacterial strains. The active ingredients that exhibited greater toxicity towards bacterial strains also displayed toxicity towards HT-29 cells (Watkins et al., 2017; Ilhan et al., 2022). Notably, previous studies have suggested that common food and drug additives, including artificial sweeteners, can alter the composition and function of the gut microbiota in batch-culture systems (Gerasimidis et al., 2020). Aspartame and benzoate have been shown to increase the relative abundance of *Bifidobacterium* in these mixed cultures, indicating a potential prebiotic effect (Gerasimidis et al., 2020). In addition, the supernatant of *Bifidobacterium longum* was found to reduce the cytotoxic effects of carbamazepine and lamotrigine. Similarly, the supernatants of *Akkermansia muciniphila* or mixed bacterial communities reduced the expression of drug resistance genes in HT-29 cell lines (Ilhan et al., 2022). In conclusion, these findings reveal that medications targeted for humans may inadvertently affect the gut microbiome, potentially exerting anti-commensal effects. This highlights the complex relationship between human-targeted medications and the gut microbiome, emphasizing the importance of understanding and modulating the microbiome to optimize treatment outcomes.

In general, the gut microbiota appears to play a significant role in the functioning and development of fundamental physiological processes, and it may also have an impact on central neural processes through the microbiota-gut-brain (MGB) axis. Both preclinical and clinical studies suggest that the microbiome could potentially modulate seizures and contribute to the pathogenesis of epilepsy. Various interventions, such as changes in diet, supplementation, and medication, have the potential to directly and indirectly affect the MGB axis. Researching the effects of these interventions could lead to a better understanding of epilepsy, the identification of biomarkers, and the development of new therapeutic options. That’s where our interest in the field lies. However, investigating the MGB axis and the role of gut supplementation in epilepsy is challenging due to the numerous potential pathways and variables involved. To date, only a few limited studies have been conducted, making it premature to draw conclusions. We also acknowledge that it is essential to conduct studies with the same rigor as pharmaceutical drug development trials, including taxa and metabolomic analyses using standard methodologies.

## 7 The association of gut microbiota with potential differences in behavioral characteristics of epileptic patients

Given the bidirectional relationship between the brain and the gut, it is important to note that neurological disorders may have a significant impact on the gastrointestinal tract. This impact can manifest in various ways, including the occurrence of sialorrhea, dysphagia, anorexia, gastroparesis, and motility disorders such as diarrhea, intestinal pseudo-obstruction, fecal incontinence, and constipation (Camilleri, 2021). Consequently, epilepsy can also affect the gastrointestinal tract in different forms, including abdominal aura, epilepsy with abdominal pain, and the adverse effects of medications on the gut and the gut microbiota.

An epigastric aura, also known as a visceral aura, is a somatosensory phenomenon often characterized by a growing sensation in the upper abdomen. This type of aura can manifest as visceral sensations, such as abdominal discomfort, visceromotor symptoms like vomiting, borborygmi, or tachycardia, as well as vegetative symptoms, including blushing or sweating. The occurrence of epigastric aura is linked to abnormal neuronal activation and discharges in the sensory cortex that represent the abdominal viscera (Schmitt and Ebner, 2000; Blom, 2010; Al-Beltagi and Saeed, 2022). Notably, this type of aura is frequently observed in epilepsy, with epigastric auras being the most common aura in medial temporal lobe epilepsy. Epilepsy with abdominal pain is a relatively rare condition characterized by recurrent paroxysms of abdominal and periumbilical pain, often accompanied by symptoms such as nausea and vomiting. This form of temporal lobe epilepsy typically presents with abdominal auras and can occur in both children and adults (Kshirsagar et al., 2012). Following these episodes, individuals may experience characteristic postictal manifestations, including lethargy, drowsiness, blindness, headache, paraesthesia, or even convulsions. For example, Remick et al. (1980) described three patients who experienced postictal hyperphagia. In some cases, postictal hyperphagia and compulsive water drinking have been reported, particularly in patients with secondary epilepsy due to temporal lobe lesions, showing a notable response to carbamazepine (Ott, 1991). Similar manifestations have also been observed in secondary epilepsy due to frontal lobe lesions (Mittal et al., 2001).

Epilepsy may have a significant impact on the microbiota, which may be due to various social, physical, and dietary differences between patients with epilepsy and normal controls. For example, patients with epilepsy may experience excessive sleepiness, have different dietary preferences due to comorbid autism, have mobility limitations, aspirate food more frequently, rely on mechanically soft diets, and consume more processed foods. Gastroesophageal reflux disease (GERD) is a common comorbidity in children with neurological problems like cerebral palsy and epilepsy. The presence of GERD in such patients can complicate their management and mimic refractory seizures, making it difficult to diagnose and treat the underlying condition (Bayram et al., 2016). Peptic ulcers are also more common in patients with epilepsy than in the general population, with some studies reporting up to eight times higher prevalence rates (Keezer et al., 2016). Similarly, the incidence of IBS is five times higher in patients with epilepsy than in controls (Camara-Lemarroy et al., 2016). Children with epilepsy also have a higher incidence of functional gastrointestinal disorders, including IBS, compared to their matched controls (Aydemir et al., 2020). Additionally, children with autism, who have a high rate of celiac disease and gut dysbiosis, are more likely to develop epilepsy. Abnormal EEG results are present in 60% of children with autism, compared to only 6%–7% of typically developed children, while epilepsy is present in 10%–30% of children with autism.

Furthermore, epilepsy may manifest with postictal states that exhibit gastrointestinal symptoms, including postictal hypersalivation, compulsive water drinking, and hyperphagia (Ott, 1991; Mittal et al., 2001; Al-Beltagi and Saeed, 2022). These postictal manifestations, particularly prevalent in patients with medication-refractory epilepsy and comorbid conditions such as cerebral palsy, have the potential to significantly impact the composition and functionality of the gut microbiota. Despite this, there remains a gap in understanding how the social, psychological, and dietary characteristics of individuals with epilepsy, either alone or in conjunction with other disorders, contribute to alterations in gut microbes. Closing this knowledge gap is crucial for developing a comprehensive understanding of the intricate interplay between epilepsy, comorbid conditions, and the gut microbiome.

## 8 Conclusion

The brain-gut-microbiota axis plays a critical role in maintaining a delicate balance between the brain and gut, primarily through visceral sensory, endocrine, and immune pathways. It is intricately involved in the pathophysiological mechanisms of gastrointestinal and neurological disorders, with a particular focus on epilepsy. In recent years, a growing body of evidence from animal models and clinical studies has highlighted the significant role of gut microbiota in epilepsy. Not only does it contribute to the occurrence of seizures through various pathways, including visceral sensory, endocrine, and immune mechanisms, but it also influences the therapeutic effects of epilepsy drugs and KD. Disparities in fecal microbial composition have been observed in both epilepsy patients and animal models before and after treatment with a KD. This underscores the increasing number of studies that are investigating the gut microbiota as a crucial factor in epilepsy. However, the current research presents a complex and sometimes contradictory picture, with discrepancies observed in the gut microbiota composition across different epilepsy models, as well as variations in clinical evidence. Furthermore, there is a lack of comprehensive mechanistic studies that would definitively establish the important role of gut microbiota in epileptogenesis and disease progression. Nevertheless, this growing body of evidence also suggests that targeting the gut microbiota, along with dietary interventions, holds significant potential for the prevention, diagnosis, treatment, and prognosis of epilepsy.
